# Interleukin-12 Delivery Strategies and Advances in Tumor Immunotherapy

**DOI:** 10.3390/cimb46100686

**Published:** 2024-10-16

**Authors:** Chunyan Dong, Dejiang Tan, Huimin Sun, Zhuang Li, Linyu Zhang, Yiyang Zheng, Sihan Liu, Yu Zhang, Qing He

**Affiliations:** State Key Laboratory of Drug Regulatory Sciences, National Institutes for Food and Drug Control, Beijing 102629, China; 13261306828@163.com (C.D.); tandj@nifdc.org.cn (D.T.); sunhm@nifdc.org.cn (H.S.); lizhuang0422@163.com (Z.L.); zly6802024@163.com (L.Z.); 15195222136@163.com (Y.Z.); 13052799837@163.com (S.L.); zhangyudw17@foxmail.com (Y.Z.)

**Keywords:** IL-12, immunotherapy, delivery system

## Abstract

Interleukin-12 (IL-12) is considered to be a promising cytokine for enhancing an antitumor immune response; however, recombinant IL-12 has shown significant toxicity and limited efficacy in early clinical trials. Recently, many strategies for delivering IL-12 to tumor tissues have been developed, such as modifying IL-12, utilizing viral vectors, non-viral vectors, and cellular vectors. Previous studies have found that the fusion of IL-12 with extracellular matrix proteins, collagen, and immune factors is a way to enhance its therapeutic potential. In addition, studies have demonstrated that viral vectors are a good platform, and a variety of viruses such as oncolytic viruses, adenoviruses, and poxviruses have been used to deliver IL-12—with testing previously conducted in various cancer models. The local expression of IL-12 in tumors based on viral delivery avoids systemic toxicity while inducing effective antitumor immunity and acting synergistically with other therapies without compromising safety. In addition, lipid nanoparticles are currently considered to be the most mature drug delivery system. Moreover, cells are also considered to be drug carriers because they can effectively deliver therapeutic substances to tumors. In this article, we will systematically discuss the anti-tumor effects of IL-12 on its own or in combination with other therapies based on different delivery strategies.

## 1. Introduction

Cytokines are important factors that regulate immune cells to effectively control the growth of tumor cells, which can directly inhibit the activity of tumor cells but also indirectly enhance the killing toxicity of tumor cells by stimulating immune cells [1]. Many cytokines have been shown to significantly inhibit the growth of various tumor cells, and IL-12 is regarded as a good cytokine to induce an antitumor immune response due to its ability to activate innate and adaptive immune responses [1,2,3,4,5]. The therapeutic potential of IL-12 has been fully validated in multiple preclinical tumor models, and its use alone or in combination with other drugs has produced promising therapeutic results, even in tumors with antagonistic immune checkpoint inhibitors (ICI) [6,7,8,9,10]. However, although IL-12 has strong anticancer effects and high in vitro activity, systemic administration of therapeutic doses is limited due to severe dose-limiting toxicity, the short half-life of conventional IL-12 drugs in vivo, and lethal off-target and off-tumor side effects [8,10,11,12]. Many times, IL-12 has been mediocre in clinical trials, which may be related to its short half-life in serum and limited activation of T cells [13]. Therefore, many methods have been proposed and explored in order to reduce the toxic events associated with IL-12 administration and improve its therapeutic effect [14,15,16].

**Figure 1:**
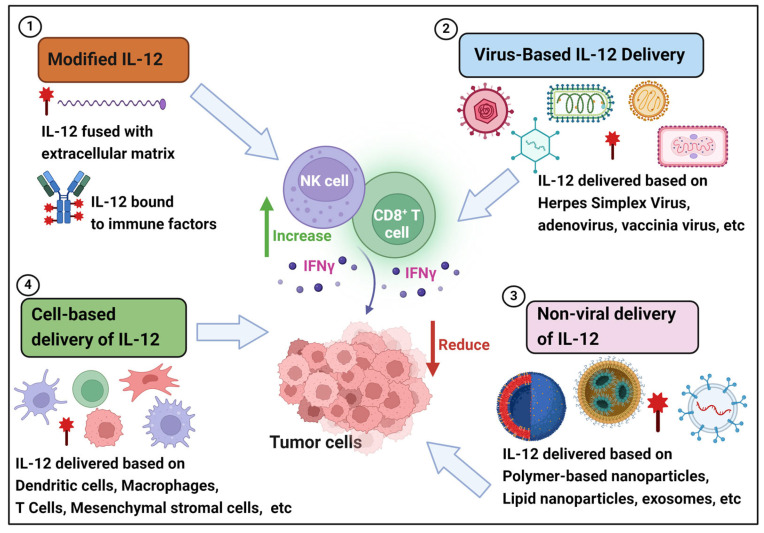
Antitumor activity of IL-12 delivered by different strategies.

Recently, the development of a new generation of IL-12 drugs focuses on reducing systemic leakage of IL-12 and increasing its safety but achieving high concentrations locally in the tumor. The modification or delivery strategies of cancer immunotherapy drugs targeting IL-12 mainly include the following categories: fusion with molecules targeting specific tumor tissues to achieve targeted delivery, application of viruses to infect tumor tissues, direct delivery of genetic material to target tissues through physical or chemical means, delivery through cellular carriers, etc. In this article, we will focus on discussing the antitumor effects of modified, virally delivered, non-viral, and cell-delivered IL-12 drugs as single or combination therapies (Figure 1).

## 2. Progress of IL-12 in Cancer Therapy Based on Different Delivery Strategies

### 2.1. Modified IL-12 for Cancer Therapy

In order to increase the possibility of IL-12 in tumor therapy, several strategies have been used to modify IL-12 to develop novel IL-12 drugs. For example, IL-12 drugs are developed by fusing with extracellular matrix proteins, collagen in the tumor microenvironment (TME), and immune factors (Table 1). Most solid tumors are encapsulated in the extracellular matrix, and targeting extracellular matrix proteins can promote the accumulation of immune cytokines in tumor tissues and reduce toxicity [17,18]. Therefore, an IL-12 drug based on this was designed, namely Pro-IL-12 [17]. In preclinical animal models, intraperitoneal (i.p.) injection of low-dose Pro-IL-12 can significantly inhibit tumor growth and prolong survival of MC38, B16F10, and 4T1 tumor-bearing mice with very low toxicity [17]. The mechanism stems from the production of tumor-specific CD8^+^ T cells and interferon-γ (IFN-γ) within the tumor [17]. In addition, the combination of Pro-IL-12 with tyrosine kinase inhibitors (TKIs) targeted therapy and ICI can further improve the therapeutic effect [17]. Similarly, IL-12–MSA–Lumican (fusion with Lumican, which specific binding of type I and IV collagen) and CBD-IL-12 (fusion collagen binding domain) were designed to be more effective and less toxic than unmodified IL-12 alone or in combination with PD-1 antibodies [19,20].

In addition, binding IL-12 to immune factors is also a strategy that has received much attention. For example, IL12-L19 (human IL-12 tandem L19 antibody) [21], IL12-scFv(L19)-FLAG (IL-12 derivatives fuse anti-EDB antibody fragment scFv(L19)) [22], and NHS-muIL12 (fuses a DNA/DNA–histone complex antibody (NHS76)) [23]. Taking NHS-muIL12 as an example, its subcutaneous (s.c.) injection has a longer half-life than recombinant IL-12, and whether used as a monotherapy or in combination with PD-L1 antibodies, it can stimulate antitumor activity, enhance cytotoxic function, and increase the production of IFN-γ and other cytokines by activating natural killer cells (NK) and CD8^+^ T cells [24,25].

**Table 1:** Modified IL-12 for cancer therapy.

Name	Manner	Cancer Model	RoA	Combination Therapy	Ref
pro-IL-12	extracellular matrix proteins	MC38, B16F10, 4T1	i.p.	/	[17]
M-L-IL-12	fused with a domain of the IL-12 receptor	EMT6, B16F10	intravenous (i.v.)	PD-1 antibodies	[18]
IL-12-MSA-Lumican	fused with Lumican	B16F10	intratumoral (i.t.)	PD-1 antibodies	[19]
CBD-IL-12	fused with collagen-binding domain	EMT6, B16F10	i.t.	PD-1 antibodies	[20]
IL12-scFv(L19)-FLAG	fused with anti-EDB antibody fragment scFv(L19)	F9	i.v.	/	[22]
mIL12-F_H_AB-hIL15	fused single-chain human IL-12 and native human IL-15 in cis onto a fully human albumin binding (FHAB) domain single-chain antibody fragment (scFv)	B16F10	i.v.	/	[26]
scIL-12-B7TM	membrane-bound IL-12 containing murine single-chain IL-12 and B7-1 transmembrane and cytoplasmic domains	CT26	i.t.	/	[27]
NHS-IL12	fused with a DNA/DNA–histone complex antibody (NHS76)	MC38, MB49, 4T1, EMT6	s.c.	Bintrafusp alfa, PD-L1 antibodies	[23,24,25]

### 2.2. Virus-Based IL-12 Delivery for Cancer Therapy

Cytokine therapy strategies based on viral system loading have demonstrated good antitumor effects in multiple tumor models by inducing local and systemic immune responses [28]. Potential viral vectors for gene delivery include the herpes simplex virus (HSV), adenovirus (AV), Vaccinia virus (VV), and other viral vectors. Next, we will focus on the progress of virus-mediated IL-12 in tumor therapy.

#### 2.2.1. Herpes Simplex Virus

HSV was the first virus to be recognized as a candidate oncolytic virus (OV), ranking highly on the list of clinical trials [29,30]. Talimogene laherparepvec (T-VEC), as the first HSV, was approved by the US FDA for advanced melanoma in 2015 [31]. With the approval of T-VEC, much research has focused on developing drugs based on HSV strategies. In preclinical studies, i.t. injections of HSV encoding IL-12 have mediated the inhibition of colon cancer [32,33,34,35,36], breast cancer [34,37], glioma [38,39,40,41,42,43,44,45], lymphoma [33,46,47], melanoma [48], and other cancers (Table 2). Indeed, most HSV-encoded IL-12 has shown good antitumor effects and prolonged survival with reduced toxicity in preclinical animal models. The mechanism is usually to change the TME, increase CD8^+^ T cell infiltration, promote IFN-γ production, and inhibit Treg function.

In addition, combination therapy is also of concern, with most studies in combination with ICI. For example, G47Δ-mIL12, G47Δ, G47Δ-mIL12, and R-123 have been explored for immunotherapy in combination with anti-PD-1 or anti-CTLA4 antibodies [38,40,41,46]. Notably, G47Δ is an HSV with deletion of γ34.5 and α47 genes, which enhances viral replication and induces the host antitumor immune response [49]. Indeed, G47Δ-mIL12, anti-CTLA4, and anti-PD-1 triple therapy showed surprising results, curing most mice in glioma models and outperforming G47Δ-mIL12, anti-CTLA4, and anti-PD-1 alone in prolonging survival [41].

**Table 2:** Viral vectors of IL-12 for cancer therapy—Herpes Simplex Virus.

Name	Dose (pfu)	Cancer Model	RoA	Combination Therapy	Ref
dvIL12-tk/tsK	2 × 10^5^	CT26	i.t.	/	[32]
VG161	5 × 10^6^	CT26, A20	i.t.	/	[33]
O-HSV12	10^7^	MC38	i.t.	/	[36]
VG2026	10^8^	A20	i.t.	/	[47]
∆6/GM/IL12	10^7^	B16F10	i.t.	/	[48]
G47Δ-mIL2	5 × 10^5^	005 GSC, CT-2A, GL261	i.t.	/	[45]
	9× 10^5^	M3 cells	i.t.	/	[50]
	2 × 10^6^	4T1	i.t.	/	[37]
	10^6^	U87	i.t.	G47Δ-mAngio	[43]
	5 × 10^5^	005 GSCs	i.t.	/	[51]
	5 × 10^5^	005 GSCs	i.t.	TMZ, d O6-BG	[39]
	2.5 × 10^5^	005 GSCs, MGG123 GSCs	i.t.	Axitinib, CTLA4 antibodies	[38]
	5 × 10^5^	005 GSCs	i.t.	PD-1, CTLA4 antibodies	[40]
	5 × 10^5^	Glioma, CT-2A	i.t.	PD-1, CTLA4 antibodies	[41]
C5252	5 × 10^6^	U87	i.t.	/	[44]
oHSV2-IL12	10^7^	4T1, CT26	i.t.	oHSV2-PD1v, IL7 × CCL19, GM-CSF and IL15	[34]
vHsv-IL-12	8 × 10^3^–2 × 10^6^	Neuro2a	i.t.	vHsv-B7.1-Ig and IL-18	[42]
NV1042	5 × 10^7^	SCC	i.v.	/	[52]
	10^7^	CT26	i.t.	/	[35]
	5 × 10^5^	CWr22	i.t.	Vinblastine	[53]
	2 × 10^7^	SCC VII	i.t.	/	[54,55]
	10^7^	TRAMP-C2, Pr14-2	i.p.	/	[56]
	10^7^	McA-R-7777	i.t.	/	[57]
M002	10^7^	Neuro-2a	i.t.	M010 (HSV expressing CCL2)	[58]
	10^7^	SARC	i.t.	/	[59]
	10^7^	X21415, D456, GBM-12, UAB106	i.t.	/	[60]
	1.5 × 10^7^	Intracranial SCK	i.t.	/	[61]
	10^7^	Xenograft SK-N-AS and SK-N-BE, Neuro-2a	i.t.	irradiation (XRT)	[62]
	10^7^	HuH6, G401, SK-NEP-1	i.t.	irradiation (XRT)	[63]
R-115	1 × 10^8^–2 × 10^9^	HER2	i.p.	/	[64]
	2 × 10^6^, 1 × 10^8^	HER2	i.t.		[65]
R-123	10^8^	HER2-LLC1	i.t.	PD-1 antibodies	[46]
T2850 T3855	10^7^	A20, MFC	i.t.	/	[66]
	5 × 10^6^ 10^7^ 3 × 10^7^	B16	i.t.	/	[66]

#### 2.2.2. Adenovirus or Adeno-Associated Virus (AAV)

AV and AAV are ideal candidate platforms, with genome stability, relative ease of manipulation, easy access to high titers, strong immunogenicity, and a wide host range [67,68,69,70,71]. Preclinical studies have tested the tumor inhibitory effect of AV or AAV vectors expressing IL-12 in various tumor models such as sarcoma [72], glioblastoma [73,74,75], prostate cancer [28], colorectal cancer [76], melanoma [77,78,79], hepatocellular carcinoma [80,81], and others (Table 3). To improve efficacy, one study designed an AV-mediated co-expression of IL-12 and the 4-1BB ligand (4-1BBL) that showed stronger antitumor effects in B16 tumor-bearing mice [78]. Similarly, studies of AV-encoded IL-12 are also exploring combinations with other therapies. Previous studies have shown that the combination therapy of adenovirus-encoded IL-12 with radiotherapy, cell therapy, or immunoblot inhibitors has a good antitumor effect in prostate cancer [82], melanoma [78,79], colon cancer [83], and lung cancer [84].

#### 2.2.3. Vaccinia Virus or Modified Vaccinia Virus (MVA)

Currently, although OVs are a promising approach, their efficacy against inaccessible and metastatic cancers—which require systematic treatment—is very limited. The VV and MVA are particularly strong OV candidates for the treatment of inaccessible and metastatic cancers because of the number of intrinsic characteristics that make it superior to other viruses in clinical development, particularly the lack of need for specific surface receptors and the ability to replicate in an oxygen-deficient environment [102,103,104,105]. Moreover, most OV delivery is limited to i.t. injection whereas VV has been reported to reach tumors after intravenous delivery [106]. The progress of IL-12 in tumor therapy with VV or MVA delivery is listed in Table 4. In one study, i.t. injection of a tumor-selective VV encoding IL-7 and IL-12 (hIL-7/mIL-12-VV) had considerable antitumor effects in B16F10, CT26, and LLC models, and even distant tumor suppression [107]. In addition, the combination of hIL-7/mIL-12-VV with anti-PD-L1 or CTLA4 antibodies showed a stronger antitumor effect in CT26 models, with complete regression in almost all mice without indications of cytokine storm despite the extent of tumor regression [107]. The combination of hIL-7/mIL-12-VV with anti-PD-L1 or CTLA4 antibodies induced tumor regression via enhancing the CD8^+^ T cells and reducing the Tregs [107].

#### 2.2.4. Other Viruses

In addition to HSV, AV, and VV, other different viral vectors—such as the Measles vaccine strain viruses (MeV), Newcastle disease virus (NDV), Semliki Forest virus (SFV), Maraba Virus (MV), Vesicular stomatitis virus (VSV), Sindbis virus (SV), Canarypox virus, and Varicella-zoster virus (VZV)—have also been modified to express IL-12, and their related research progress is summarized in Table 5.

### 2.3. Non-Viral Delivery of IL-12 for Cancer Therapy

Many early studies used viruses as delivery vectors; however, serious clinical adverse events caused by the potential carcinogenicity and high immunogenicity of some viral vectors have affected their application in clinical trials. With the continuous development of materials and preparation technologies, non-viral vectors (such as bio-derived materials and chemical materials) with low cost, easy synthesis, easy purification, high transfection efficiency, and low immunogenicity have become the main candidates for drug delivery. In chemical-based delivery systems, polymer-based nanoparticles and lipid-based nanoparticles are widely used due to their advantages of high efficiency and diversity [148]. Bio-derived vectors, which mainly include exosomes (Exos) [149,150,151], bacterial outer membrane vesicles (Omv) [152,153], and virus-like particles (VLPs) [154,155], have been shown to be attractive in some applications. In this section, we will describe each delivery system and highlight its application in cancer therapy for delivering IL-12.

#### 2.3.1. Chemical-Based Delivery Systems

Polymer-based nanoparticles

Polymers, which are generally spherical particles formed by electrostatic interactions of polymer molecules with negatively charged nucleic acids, have been extensively studied and reviewed as delivery systems for DNA- and RNA-based drugs. Polymeric vectors mainly include the following: (1) Poly(ethyleneimine) (PEI). (2) Poly (amino acid) s, such as P(Lys), P(Orn), or P(Asp). (3) Polyesters including PLGA (Poly (lactic-co-glycolic acid)), PBAEs (poly (β-amino ester) s) and PACE (polyplexes based on poly(amine-co-ester)). (4) Natural polymers include chitosan and Protamine. (5) Other polymers and dendrimers include the RAFT polymer (DMAEMA), the dendrimer PAMAM, as well as others [156,157,158,159,160]. Polymer-based strategies are believed to have a strong ability to deliver different nucleic acids by preventing nucleic acid degradation as well as promoting cellular uptake and endosomal escape [160]. In this section, we will highlight the latest applications of polymers-delivered IL-12 in cancer therapy.

In preclinical models, polymer-based IL-12 has been used to treat melanoma, colon cancer, liver cancer, breast cancer, lung cancer, ovarian cancer, and others (Table 6). Among them, PBAEs are considered a safe alternative to nucleic acid delivery due to their biodegradability. In one study, Neshat et al. engineered biodegradable lipophilic PBAE delivery with the co-stimulatory signaling molecules 4-1BB ligand (4-1BBL) and soluble IL-12 (4-1BBL +IL-12 NPs) which, in combination with PD-1 antibodies, effectively induced tumor regression and clearing and resistance to distant tumor reattack [161]. In addition, a study developed a co-delivery system that delivers cisplatin (CDDP) and plasmids encoding the IL-12 gene (HC/pIL-12/polyMET), acting synergistically through chemotherapy sensitization and microenvironment regulation [162]. HC/pIL-12/polyMET has an ideal particle size, superior serum stability, effective intracellular CDDP release, and IL-12 transfection efficiency [162]; more importantly, the long-circulating HC/pIL-12/polyMET micelle clusters promoted the accumulation of CDDP and IL-12 at the tumor site, thus significantly inhibiting the growth of tumor and prolonging the overall survival of LLC tumor-bearing mice [162]. HC/pIL-12/polyMET has a synergistic chemoimmunotherapy effect by increasing IFN-γ released by immune effector cells and inducing differentiation of tumor-associated macrophages into type M1 [162]. In another study, a novel polymetformin (PMet)-based nanosystem that co-delivers doxorubicin (DOX) and a plasmid encoding the IL-12 gene (HA/pIL-12/DOX-PMET) was developed for the treatment of metastatic breast cancer [163]. HA/pIL-12/DOX-pmet extends its time in the blood circulation through tumor-specific targeting mediated by CD44 receptors, effectively accumulates in tumors, and is internalized in tumor cells [163]. HA/pIL-12/DOX-PMet micelle clusters synergically enhance NK cells and tumor-infiltrating cytotoxic T lymphocytes, regulate the polarization of tumor M2 macrophages to activated antitumor M1 macrophages, and reduce Treg cells [163]. The results showed high antitumor and antimetastatic activity in 4T1 breast cancer lung metastasis mouse models [163]. In conclusion, co-delivery nanoparticles based on multiple molecules have the dual advantages of chemotherapy and gene therapy, and co-delivery combination therapy will have great prospects in cancer therapy.

Lipid nanoparticles (LNPs)

Lipid-based delivery tools, including LNPs and lipoplexes, are the most clinically advanced platforms for mRNA delivery [164]. Currently, three RNA-LNPs have been approved by the US Food and Drug Administration (FDA) and the European Medicines Agency (EMA), namely patisiran, BNT162b2, and mRNA-1273. Patisiran is a small interfering RNA (siRNA)-LNP that treats hereditary transthyretin-mediated (hATTR) amyloidosis, and BNT162b2 and mRNA-1273 are two mRNA-LNP-based COVID-19 vaccines [165]. In order to improve the application of mRNA-LNP technology in cancer therapy, many studies are devoted to exploring and developing more efficient delivery methods, such as designing and screening novel lipid molecules, adjusting the proportion of lipids in LNPs, modifying the surface of LNPs, and selecting different delivery routes [166,167,168].

Next, we summarize the application of LNP-based IL-12 in cancer therapy (Table 6). For example, an LNP delivers IL-12 mRNA (IL-12-LNP), which can significantly reduce HCC tumor growth, delay tumor progression, and prolong survival without animal toxicity after weekly i.v. injection [169]. The mechanism is attributed to the increased infiltration of CD3^+^CD4^+^CD44^+^ immune cells but the TME has not been thoroughly explored and elaborated [169]. Compared with i.v. injection, i.t. injection of IL-12 mRNA can effectively promote the localization and sustained production of IL-12 in the TME and reduce the systemic effect [170]. mIL-12 mRNA, an LNP formulation containing mouse IL-12 mRNA, which was well tolerated, especially with less than 10% weight loss detected at the dosage levels of 0.05 and 0.5mg [170]. A single intratumoral dose of mIL12 mRNA induced regression of multiple tumors such as MC38-sensitive (MC38-S), B16F10, and A20, and even showed good antitumor effects on MC38-resistant (MC38-R) tumor models with ICI antagonism [170]. The antitumor activity of mIL12 mRNA depends on induced IFN-γ and CD8^+^ T cells and does not require NK, natural killer T cells (NKT), and its antitumor activity is also associated with TH1 TME transformation [170]. In addition, there are many studies exploring IL-12 in combination with other therapies. Local mIL12 mRNA induces a systemic antitumor immune response to distal lesions and exhibits a synergistic tumor suppressive effect in combination with PD-L1 antibody therapy [170]. Since IL-12 has shown promising results in combination with other therapies, direct delivery of IL-12 and other target mRNAs may also have exciting results. F-PLP/pIL12, an FRα-targeted IL-12 lipoplex, has tumor-cell targeting and IL-12 delivery functions [171]. For folate receptor α (FRα) overexpression in colon cancer, F-PLP/pIL12 treatment significantly inhibits CT26 tumor growth and is safe, accompanied by increased IL-12 expression and IFN-γ secretion in tumor tissues [171]. The antitumor mechanisms include inducing tumor cell apoptosis, reducing microvascular density, stimulating TNF-α secretion, and activating NK [171]. Therefore, dual-targeted or even multi-targeted IL-12 lipid nanoparticles may be a promising platform for cancer immunotherapy in the future.

**Table 6:** Chemical-based delivery systems.

Name	Carrier Description	Cancer Model	RoA	Combination Therapy	Ref
**Polymer-based nanoparticles**					
PEI:IL-12	polyethylenimine (PEI)	osteosarcoma	aerosol	/	[172]
PEI-IL12	PEI-DNA nanoparticles carrying IL12 gene	LLC, CT26	i.v.	/	[173]
mIL-12	polyethylenimine (PEI)	osteosarcoma	intranasal (i.n.)	/	[174]
IL-12	ifosfamide (IFX) with or without intranasal polyethylenimine (PEI)	LM7 osteosarcoma	i.n.	ifosfamide	[175]
mIL-12	poly[α-(4-aminobutyl)-L-glycolic acid] (PAGA)	CT26	i.t.	/	[176]
p2CMVmlL12	poly-(D,L-lactic-co-glycolic acid) (PLGA) microspheres	CT26	s.c.	/	[177]
pmIL-12	poly[alpha-(4-aminobutyl)-L-glycolic acid] (PAGA)	CT26	i.t.	/	[178]
4-1BBL and IL-12 mRNA	biodegradable, lipophilic poly (beta-amino ester) (PBAE) nanoparticles	E0771, MC38	i.t.	PD-1 antibodies	[161]
HC/pIL-12/polyMET	HC/pIL-12/polyMET micelleplexes	LLC	i.v.	/	[162]
HA/pIL-12/DOX-PMet	HA/pIL-12/DOX-PMet micelleplexes	4T1	i.v.	/	[163]
p2CMVmIL-12	water-soluble lipopolymer (WSLP)	CT26	i.t.	/	[179]
p2CMVmIL-12	water-soluble lipopolymer (WSLP)	4T1, EMT6	i.t.	paclitaxel	[180]
p2CMVmIL-12	water-soluble lipopolymer (WSLP)	4T1	i.t.	paclitaxel	[181]
p2CMVmIL-12	water-soluble lipopolymers using cholesteryl chloroformate (WSLP) and PEI	CT26	i.t.	/	[182]
IL-12 plasmid	puly(N-lnethyldietheneamine sebacate) (PMDS) and cholesterol	4T1	i.t.	/	[183]
pmIL-12	mannosylated chitosan	CT26	i.t.	/	[184]
pmIL-12	polyethylenimine covalently modified with methoxypolyethyleneglycol and cholesterol	GL261	Intracranial (i.c.)	carmustine	[185]
pCMV IL-12	poly (D,L-lactic-co-glycolic) acid (PLGA) (50:50) with the cationic lipid 1,2-dioleoyl-3-(trimethylammonium) propane (DOTAP) and the ligand asialofetuin (AF)	BNL	i.t.	/	[186]
CPP-IL-12	CaCO3-polydopamine-polyethylenimine (CPP)	B16F10	i.t.	/	[187]
Nano-IL-12	carboxydimethyl-maleic anhydride (CDM)-modified poly(ethylene glycol)-poly(L-Lysine) (PEG-pLL(CDM))	4T1 TNBC, B16F10	i.v.	CTLA4 and PD-1 antibodies	[188]
TINPs	dual-target PLGA nanoparticles	HepG-2	/	/	[189]
**Lipid nanoparticles**					
IL-12-LNP	lipid nanoparticles (LNPs)	HCC	i.v.	/	[169]
IL12 mRNA	a novel lipid nanoparticle (LNP)	MC38, B16F10, A20	i.t.	PD-L1 antibodies	[170]
F-PLP/pIL12	an FRα-targeted lipoplex	CT26	i.p.	/	[171]
DAL4-LNP-IL-12 mRNA and IL-27 mRNA	ionizable lipid materials containing di-amino groups with various head groups (DALs)-DAL4-LNP	B16F10	i.t.	/	[190]
JCXH-211	lipid-nanoparticle-encapsulated self-replicating RNA (srRNA) encoding IL-12	MC38, B16F10, EMT6	i.v. i.t.	PD-1 antibodies	[191]
LNP-Rep(IL-12-alb)	lipid nanoparticles (LNPs)	B16F10, CT26	i.t.	PD-1 antibodies	[192]
IL-12 mRNA	calcium carbonate nanoparticles	GL261	i.v.	ultrasound	[193]
IL12LNP	lipid nanoparticles (LNPs)	HT29	i.t.	/	[194]
IL-12 circRNA LNP	ionizable lipid nanoparticles	LLC1	i.t.	PD-L1 antibodies	[195]
pCMVIL-12	transferrin (Tf)-lipoplexes	CT26	i.t.	/	[196]
DMP/IL-12	monomethoxy poly (ethylene glycol)–poly (caprolactone) with the DOTAP lipid	C26, LL/2	i.p.	/	[197]
ATRA–cationic liposome/IL-12 pDNA	all-trans-retinoic acid (ATRA)-incorporated cationic liposome (ATRA–cationic liposome)	colon26 cells	i.v.	/	[198]

#### 2.3.2. Bio-Derived Delivery Vector

Extracellular vesicles (EVs) are important intercellular communication systems that promote the transfer of macromolecules between cells. For example, Exos are considered as natural mRNA delivery systems. In one study, an inhalable extracellular vesicle loaded with IL-12 mRNA (IL-12-Exo) was developed, which effectively controlled the development of lung cancer and enhanced systemic immunity [199]. Importantly, the specific targeting of IL-12-Exo to lung tumors is much higher than that of liposome loaded with IL-12 mRNA (IL-12-Lipo) by about 1.54 times [199]. IL-12-Exo significantly inhibited LL/2 and B16F10 tumor growth and progression, accompanied by moderate weight gain in mice [199]. The antitumor mechanism was attributed to the remodeling of TME, increasing the proportion of CD8^+^ T cells, CD4^+^ T cells, NK, and NKT populations, while decreasing the proportion of immunosuppressive Tregs and myeloid-derived suppressor cells (MDSCs) [199]. IL-12 mRNA stimulates upregulation of a broad spectrum of inflammatory cytokines and chemokines in TME, especially the sustained secretion of high levels of IFN-γ [199]. Compared with liposome delivery systems, exosomes enhance the expression of IL-12 and greatly reduce its toxicity in vivo [199]. As a non-invasive method, inhalation is expected to lead to better patient compliance than i.t. injection. Exos, as biocompatible vesicles, provide a universal RNA delivery scheme [199] (Table 7).

### 2.4. Cell-Based Delivery of IL-12 for Cancer Therapy

Recently, cells have also been proposed as drug carriers because of their effective delivery of therapeutic substances to the tumor [204,205,206,207]. Dendritic cells (DCs), T cells, mesenchymal stromal cells (MSCs) and other cells have been designed to express IL-12 for cancer therapy.

#### 2.4.1. Dendritic Cells

DCs are the most powerful antigen-presenting cells (APCs) in vivo, linking innate and adaptive immunity. DC-based therapeutic strategies have proven to be a positive approach to treating cancer by altering the TME and enhancing the systemic host immune response [208]. Following i.t. or a peritumorally (p.t.) injection, DCs have been engineered to express IL-12 to induce a powerful antitumor immune response (Table 8).

In preclinical studies, DCs transfected with IL-12 effectively inhibited the growth of various tumors such as melanoma [209,210,211,212,213,214], colon cancer [215,216,217,218,219], and other tumors [220,221,222]. The therapeutic benefits of DC-expressed IL-12 are attributed to the induction of specific CD8^+^ T cells and durable antitumor immunity. In a clinical study, the feasibility, safety, and antitumor activity of DC-transduced IL-12 (AFIL-12) in the treatment of metastatic gastrointestinal cancer was validated. The results showed that two patients were stable and eight progressed, of which two progressed rapidly during treatment, demonstrating that i.t. injection of AFIL-12 for the treatment of metastatic gastrointestinal malignancies is feasible and well tolerated but further studies are needed to determine and improve clinical efficacy [223].

**Table 8:** Cell-based delivery of IL-12 for cancer therapy—Dendritic Cells.

Name	Cancer Model	ROA	Ref
DC.RheoIL12	B16	i.t.	[209]
DC-mIL-12	B16F10	i.t.	[210]
mIL-12	B16	i.t.	[211]
DC+IL-12	Melanoma B6	i.t.	[212]
DC.IL12	B16	i.t.	[213]
gp100+IL12/DCs	B16BL6	intradermal (i.d.)	[214]
DC/IL-18+IL-12/TAg	MC38	p.t.	[216]
AdCMVmIL-12	CT26	i.t.	[215]
BM-derived DC infected with AdCMVIL-12	CT26, MC38	i.t.	[217]
AdIL12/IL18DC	CMS4, MethA	i.t.	[218]
AdIL12DC	CMS4	i.t.	[219]
mIL-12	TBJ-NB	i.t.	[220]
DC/IL-12	178-2 BMA	i.t.	[221]
DC-IL-12	RENCA	i.t.	[222]
AFIL-12	pancreatic, colorectal, primary liver, gastrointestinal cancer malignancies	i.t.	[223]

#### 2.4.2. T Cells

T-cell therapy is a promising therapeutic approach but it is often hampered by the highly immunosuppressive TME, such as limited T-cell trafficking, persistence, and durable antitumor activity. Engineering T cells to express IL-12 has been shown to improve antitumor efficacy and reduce systemic toxicity in solid tumors (Table 9). In multiple models, injection of IL-12-expressing T cells induced regression of many tumors, including melanoma [224,225,226,227,228], sarcoma [227], colorectal adenocarcinoma [227,229], and other cancers [230,231,232,233,234]. The efficacy of T-cell therapy generally depends on increasing chemokines and cytokines, promoting the proliferation of CD8^+^ T cells, and reducing the proportion of Treg cells, which can directly promote the effective enrichment of T cells and antitumor effects.

#### 2.4.3. Mesenchymal Stromal Cells

MSCs have been used in many trials due to their immunosuppressive properties and their tendency to target cancer cells, including as IL-12 vectors for solid tumors (Table 10). For example, one study observed that after i.v. administration of MSC-loaded IL-12 (MSC/IL-12) in tumor-bearing mice, tumor growth was inhibited, the number of metastases significantly decreased, blood vessel density decreased, and the number of anticancer M1 macrophages and CD8^+^ T lymphocytes in the tumors increased, without systemic toxicity [237]. In addition, Park et al. designed mesenchymal stem cells (MSC_IL-12) with glioblastoma propensity to secrete IL-12 and evaluated that MSC_IL-12 has a good efficacy in glioblastoma (25.0% cure rate) [238]. Tumor-infiltrating lymphocytes (TILs) analysis showed that MSC_IL-12 treatment resulted in CD4^+^ T cell and NK cell infiltration as well as reduced Tregs frequency [238]. Moreover, the combination of PD-1 antibodies and MSC_IL-12 showed a better antitumor effect (50% cure rate) [238]. Excitingly, no tumor growth was observed in the cured mice after re-attack, indicating long-term immunity to treatment-induced glioblastoma [238].

#### 2.4.4. Other Cells

In addition to the commonly used cell carriers such as DCs, T cells, and MSCs, there are other different cell delivery carriers, such as macrophages, NK cells, glial cells, etc., and even genetically engineered tumor cells (Table 11). In one study, autologous tumor cell vaccines via EBV/liposomes were designed to secrete IL-12 and IL-18 (B16/mIL-12+mIL-18), and repeated immunization showed strong tumor inhibition in a B16 melanoma model, accompanied by high IFN-γ production [250].

## 3. Clinical Perspectives

IL-12 with different strategic loads has been shown to have good broad-spectrum antitumor effects and safety in preclinical models, suggesting that IL-12 is an attractive therapeutic candidate. In general, safety remains the most concerning aspect when transferring results from the laboratory to the bedside, as does dose, route of administration, viral pharmacokinetics, and host cell resistance mechanisms. Currently, IL-12 is being tested in clinical studies against various cancers, such as breast cancer, lung cancer, pancreatic cancer, ovarian cancer, colorectal cancer, melanoma, etc. (Table 12). In addition, because traditional recombinant human IL-12 has been associated with different degrees of adverse reactions in clinical trials, different strategies of delivery of IL-12 are under clinical study.

The safety and antitumor efficacy of NHS-IL12, either as a single agent or in combination with other therapies, has been extensively demonstrated in many preclinical tumor models [23,24,25]. Excitingly, safety and antitumor efficacy of human NHS-IL12 was found in a Phase I clinical trial (NCT01417546) [256] to have good treatment tolerance, enhanced immune-related activity, and increased immune infiltration in TME [257]. Additionally, multiple clinical trials (NCT04287868 [258], NCT04491955 [259], NCT02994953 [260], NCT04303117 [261], and NCT04235777 [262]) have demonstrated promising results for NHS-IL12 in advanced HPV-associated malignancies, small bowel and colorectal cancers, Kaposi’s sarcoma, urothelial cancer, etc. For example, the Phase II clinical trial (NCT04491955 [259]) of the NHS-IL12 combination therapy in patients with small intestine and colon cancer showed encouraging results (CR 12.5%). Similarly, a virus expressing human IL-12 is also under clinical trial investigation as monotherapy or combination therapy in prostate cancer (NCT02555397 [263], NCT00406939 [264]), pancreatic cancer (NCT03281382 [265]), breast cancer (NCT00849459 [266], NCT00301106 [267]), melanoma (NCT01397708 [268], NCT00003556 [269]), pediatric brain tumor (NCT03330197 [270]), glioblastoma (NCT02026271 [271], NCT03636477 [272], NCT05084430 [273]), and other solid tumors (NCT04613492 [274],). In addition, electroporation is a non-viral gene delivery method of plasmid DNA. The plasmid gene encoding IL-12 in intratuminal metastasis has been proven to be safe and effective in clinical experiments and has good local tumor control effect (Table 12). In addition, the strategy of cells as carriers for delivering IL-12 has also been validated in clinical trials (Table 12). In our view, many preclinical and clinical studies of IL-12 delivered with different strategies have shown exciting performance in combination with other therapies; therefore, the combined study of IL-12 and ICI is worthy of clinical investigation and may become an attractive treatment strategy for cancer patients. It is worth mentioning that many preclinical studies have shown that liposome-loaded IL-12 mRNA has good antitumor effect and safety and its related studies deserve attention and push to clinical trials.

## 4. Conclusions and Future Directions

In the field of tumor therapy, although IL-12 is a high-profile molecule, its therapeutic effect on solid tumors is not ideal and even causes serious adverse reactions. The focus of current research is mainly on local targeted drug delivery to reduce adverse reactions, and to play a synergistic role in combination with chemoradiotherapy or immunotherapy. For systematic administration, the focus is on reducing the off-target effect of IL-12. In order to achieve the goal of IL-12 in effectively treating tumors, many strategies to modify or deliver IL-12 are under investigation. For example, studies have attempted to achieve targeted delivery of IL-12 by fusing it with sites that target specific tumor tissue. Pro-IL-12 [17], IL-12-MSA-Lumican [19], CBD-IL-12 [20], M-L-IL-12 [18], and NHS-muIL12 [23]—designed based on fusion extracellular matrix or immune molecular strategies—showed good antitumor effects in preclinical tumor-bearing mice and had synergistic effects in combination with other therapies. Among them, NHS-IL-12 has shown promising results in clinical trials for advanced HPV-associated malignancies, small bowel and colorectal cancers, Kaposi’s sarcoma, urothelial cancer, etc. (Table 12). Although IL-12 fusion with extracellular matrix or immune factors helps to prolong half-life and reduce off-target effects, systemic administration cannot completely avoid IL-12-related toxicity. In addition, the direct delivery of IL-12 to tumor tissues through viral or non-viral vectors to achieve local high-concentration delivery is also an important method. Many preclinical studies have confirmed that IL-12 delivered by virus vectors, such as the Vaccinia virus, adenovirus, and herpes virus, plays a good antitumor role. Multiple viral strategy-based delivery of IL-12 has reached clinical trials (Table 12) to demonstrate that this approach is feasible but it also needs to be continuously improved and optimized to continuously improve its antitumor ability without increasing its toxicity. Although virus-based delivery strategies have relatively good transfection efficiency, there are problems with neutralizing the immune response, high heterogeneity, susceptible normal tissue, and risk of gene integration [92,275,276,277]. Non-viral carrier polymer nanoparticles, lipid nanoparticles, and biological vesicles are also good ways to deliver IL-12 and relevant studies have performed well in preclinical studies. IL-12 delivered by this strategy has also entered clinical trials for the treatment of epithelial ovarian cancer, fallopian tube cancer, primary peritoneal cancer, etc. (Table 12). Non-viral vectors have advantages such as the cytotoxicity, immunogenicity, and mutagenicity being low but they also have limitations such as instability and easy inactivation of the carrying substances [278,279,280,281]. Moreover, nanoparticle-delivered IL-12 nucleic acid has the problem of expression efficiency and unregulated gene products. Furthermore, many cells are also engineered to express IL-12, such as transduced DCs, T cells, mesenchymal stromal cells, tumor cells, and macrophages, which are injected directly or systematically into tumors. Numerous preclinical and clinical studies have used IL-12 transduced cells for cancer therapy (Table 11 and Table 12). The administration of IL-12 transduction cells is relatively well-tolerated but there are limitations such as poor cell production, poor transduction control and reproducibility, easy in vivo rejection, and patient heterogeneity [8]. It is believed that with the in-depth study of the antitumor mechanism of IL-12 and the improvement of the IL-12 delivery strategy, drug use, pathway, and synergistic drug use, IL-12 will be successfully developed into an anticancer drug with important clinical application value.

## Figures and Tables

**Table 3:** Viral vectors of IL-12 for cancer therapy—Adenovirus or Adeno-Associated Virus.

Name	Dose (pfu)	Cancer Model	RoA	Combination Therapy	Ref
AdmIL-12	10^8^	RM-9	i.p.	/	[28]
murine IL-12	2.5 × 10^8^	Renca cells	i.t.	/	[85]
AAV9.RS-mIL-12	2.5 × 10^10^ vg/kg	Hepa1-6	i.v.	/	[86]
Ad-RTS-mIL-12	5 × 10^9^ vp	GL-261	i.t.	/	[87]
Ad-ΔB7/IL12/GMCSF	5 × 10^7^	B16F10	i.t.	/	[88]
AdV5-IL-12	1.5 × 10^8^	EMT6-HER2	p.t.	/	[89]
Ad.mIL12	/	GL261	i.t.	/	[73]
AdRGD-IL12	2 × 10^7^	Meth-A	i.t.	/	[72]
AdCMVIL-12	10^8^ and 10^9^	CT-26 cells	i.t.	/	[76]
ADV/mIL-12	3 × 10^8^	MCA-26	i.t.	/	[77]
oAd+DC	2 × 10^10^	LLC	i.t.	/	[84]
rAAV/IL-12	10^11^ vp	DBTRG	i.t.	/	[74]
rAAV2/IL12	1.96 × 10^12^	RG2	i.t.	/	[75]
AAV8-Tetbidir-Alb-IL-12	5 × 10^11^ vg/kg	MC38	i.v.	/	[70]
AAV8/IL-12	10^9^–10^11^	BNL HCC	i.v.	/	[80]
OAV-scIL-12-TM	2.5 × 10^8^ 10^9^ iu	HaP-T1	i.t.	/	[90]
Ad-DHscIL12	10^7^ iu	H2T	i.t.	/	[91]
Ad.IL-12	2.5 × 10^10^–3 × 10^12^ vp	advanced pancreatic, colorectal, or primary liver malignancies	i.t.	/	[92]
RdB/IL-12/IL-18	10^8^	B16F10	i.t.	/	[93]
YKL-IL12/B7	5 × 10^8^	B16F10	i.t.	/	[94]
AdCMVIL-12	7.5 × 10^7^	CT26	i.t.	/	[95]
Ad-IL-12	10^9^	PyMidT	i.t.	/	[96]
Ad.mIL-12	3.3 × 10^9^	7500 RM-1	i.t.	/	[97]
GL-Ad/RUhIL-12	3 × 10^9^ iu	MC-38	i.v.	RU486	[98]
Ad/IL-12	10^9^	BNL cells	i.t.	GM-CSF	[99]
AdmIL-12	10^8^–10^9^	178-2 BMA	i.t.	radiation therapy	[82]
AdIL-12	2.5 × 10^9^	Hepa129	i.t.	AdK1-3	[81]
HC-Ad/RUmIL-12	2.5 × 10^8^ iu	MC38	Intrahepatic	Oxaliplatin	[83]
Adv.mIL-12	3.2 × 10^8^	MCA26	i.t.	4-1BB antibodies	[100]
Ad5-ZD55-CCL5-IL12	10^9^	OSRC-2	i.t.	CA9-CAR-T	[101]
Ad-ΔB7/IL-12/4-1BBL	5 × 10^9^	B16F10	i.t.	dendritic cells	[78]
Ad-ΔB7/IL12/GMCSF	5 × 10^10^	B16F10	i.t.	dendritic cells	[79]

**Table 4:** Viral vectors of IL-12 for cancer therapy—Vaccinia virus or Modified Vaccinia virus.

Name	Dose (pfu)	Cancer Model	RoA	Combination Therapy	Ref
rVV–mIL-12	10^5^–10^7^	C6 glioma	i.t.	/	[108]
rVV-p53/rVV-2-12	2 × 10^7^	C6 glioma	i.t.	/	[109]
VVΔTKΔN1L-IL12	10^8^	LLC, LY2, DT6606,4T1, CT26, SCCVII, HCPC1	i.t.	/	[110]
VAC-2-12	10^7^	CT26.CL25	i.v.	/	[111]
rVVHA-IL-12	5 × 10^6^	AE17	i.t.	/	[112]
hIL-7/mIL-12-VV	2 × 10^7^	B16F10, CT26, LLC, TRAMP-C2	i.t.	PD-1 or CTLA4 antibodies	[107]
VV-IL-12mCLTX-HiBiT	10^7^ 10^8^	U2OS, ID8, 4T1.2, MC38	i.t.	PD-1 antibodies	[113]
vvDD-IL-12	10^9^	MC38, B16, AB12, CT26	i.p.	PD-1 antibodies	[114]
VACV muIL-12	10^7^	CT26, MC38	i.t.	PD-L1 antibodies	[115]
MVA-IL-12	6 × 10^5^	MC38, B16F10, CT26	i.t.	PD-1 antibodies	[116]
MVA.scIL-12	5 × 10^7^	MC38, CT26	i.p.	PD-L1 antibodies	[117]

**Table 5:** Viral vectors of IL-12 for cancer therapy—other viruses.

Name	Dose (pfu)	Cancer Model	RoA	Combination Therapy	Ref
**Measles vaccine strain viruses (MeV)**					
FmIL-12	5 × 10^5^ ciu	MC38cea, B16hCD46	i.t.	/	[118]
FmIL-12	/	MC38cea	i.t.	/	[119]
**Newcastle disease virus (NDV)**					
rAF-IL12	2^7^ HA	CT26	i.t.	/	[120]
rClone30s-IL12	10^7^	H22	i.t.	/	[121]
rAF-IL12	/	HT29	i.t.	/	[122]
**Semliki Forest virus (SFV)**					
SFV-IL12	10^7^ iu	B16	i.t.	/	[123]
rSFV/IL12	10^6^ iu	P815	i.t.	/	[124]
SFV-IL12	10^8^ vp	MC38 or TC-1	i.v.	/	[125]
IL-12 VLPs	5 × 10^8^	RG2	i.t.	/	[126]
SFV-IL12	10^8^ vp	B16, MC38, 4T1 cells	i.t.	PD-1 antibodies	[127]
SFV-IL-12	10^8^	B16, TC-1	i.t.	CD137 antibodies	[128]
SFV-IL12	10^8^	203-glioma cells	i.t.	/	[129]
rSFV10-E-IL12	4 × 10^9^ iu	CT26, 4T1	i.t.	/	[130]
SFV-IL-12	10^8^ vp	MC38	i.t.	/	[131,132]
SFV-IL-12	10^8^ vp	HCC	i.t.	/	[133]
SFV-enhIL-12	1.2 × 10^10^	HCC	i.t.	/	[134]
LSFV-IL12	10^7^–10^9^	Panc-1	i.t.	/	[135]
SFV-IL-12	2 × 10^8^ vp	4T1	i.t.	/	[136]
**Maraba Virus (MV)**					
MG1-IL12-ICV	10^5^	CT26	i.p.	/	[137]
**Vesicular stomatitis virus (VSV)**					
rVSV-IL12	10^7^	SCC	i.t.	/	[138]
rVSV-mIL12-mGMCSF	10^7^ TCID50	B16F10	i.t.	/	[139]
**Sindbis virus (SV)**					
Sin/IL12	10^7^	ES-2	i.p.	/	[140]
Sindbis/IL-12	10^7^	ES-2, MOSEC	i.p.	/	[141]
SV.IgGOX40.IL-12	5 × 10^6^ TU	MOSEC	i.p.	/	[142]
SV.IL12	5 × 10^6^ TU	CT.26	i.p.	OX40 antibodies	[143]
**Canarypox virus**					
ALVAC-IL-12	1–4 × 10^6^ TCID50	Metastatic Melanoma	i.t.	/	[144,145]
ALVAC-IL12.	2.5 × 10^5^ TCID50	TS/A	i.t.	/	[146]
**Varicella-zoster virus (VZV)**					
Ellen-ΔORF8-tet-off-scIL12	10^5^	B16F10	i.t.	/	[147]

**Table 7:** Bio-derived delivery systems.

Name	Source	Dose	Cancer Model	RoA	Ref
IL-12-Exo	human embryonic kidney cell-derived exosomes	2 × 10^9^ particles	LL/2, B16F10, 4T1	Inhal	[199]
ITGB1−mscIL12+HN3+Deg EVs	HEK293-derived EVs	5 × 10^10^ particles	Hepa1-6-hGPC3	i.v.	[200]
Tex MC38/IL12shTGFβ1	MC38-derived particles	2 × 10^6^ Particles	MC38	p.t.	[201]
exoIL-12	HEK293SF-3F6	100 ng	B16F10, MC38, CT26	i.t.	[202]
IL-12-encapsulated DEVs (DEV-IL)	mature dendritic cells (DEVs)	25 μg	GL-261	s.c.	[203]

**Table 9:** Cell-based delivery of IL-12 for cancer therapy—T cells.

Name	Cancer Model	ROA	Ref
OT-I-IL-12	B16-OVA, PANC02-OVA	i.p.	[224]
OT1-IL-12 mRNA	B16-OVA	i.t.	[225]
IL-12 + DRIL18	B16-OVA	i.t.	[226]
IL-12	B16 tumors	i.v.	[228]
DC101 CAR-Flexi-IL12	B16F10, MCA205, MC17-51, MC38, CT26	i.v.	[227]
T cells CAR+iIL-12	CEA^−^ MC38, CEA^+^ C15A3	s.c.	[229]
mIL12 and mIFNα2	GL-261, CT-2A, SMA-560	i.v.	[230]
19mz/IL-12	EL4	i.v.	[231]
CAR-IL12 T-cells	A20	i.v.	[232]
4H11-28z/IL-12	SKOV3	i.p.	[233]
GPC3-28Z-NFAT-IL-12	PLC/PRF/5, Huh-7	i.v.	[234]
INS-CAR T	Raji	i.v.	[235]
RB-312	HT1080, FaDu	i.t.	[236]

**Table 10:** Cell-based delivery of IL-12 for cancer therapy—Mesenchymal Stromal Cells.

Name	Cancer Model	ROA	Ref
MSC/IL-12	B16F10	i.t.	[237]
MSC/IL-12	B16F10	i.p.	[239]
MSC(IL-12)	glioblastoma GL26	i.t.	[238]
CAd12_PD-L1 MSCs	A549, H1650	i.v.	[240]
IL-12 MSCs	4T1	s.c.	[241]
MSC-AdIL12	Ast11.9-2	/	[242]
MSC/IL-12	786-0	i.v.	[243]
MSCs/IL-12	HCa-I, Hepa 1-6	i.t.	[244]
FYD + IL-12 + BMSCs	U251	i.v.	[245]
MB/IL12-MSCs	EMT6	i.v.	[246]
CAR+MSC IL7/IL12	LS174T	s.c.	[247]
MSCs/IL-12M	B16F10	i.t.	[248]
UCB-MSC-IL12M	GL26	i.t.	[249]

**Table 11:** Cell-based delivery of IL-12 for cancer therapy—other cells.

Name	Cell Type	Cancer Model	ROA	Ref
AdmIL-12	Macrophages	178-2BMA	i.t.	[251]
G/M//AdmIL-12	Macrophages	178-2BMA	i.t.	[252]
GD2.CAR(I)IL12	Human natural killer T cells	BV-173, CHLA-255	i.v.	[253]
B16/mIL-12+mIL-18	Autologous tumor cells	B16	s.c.	[250]
Neuro2a/IL-12/IL-15	Neuro2a cells	neuroblastoma	i.v.	[254]
pT-mIL12 and pCMV-m7pB	OT-I cells	B16/OVA	i.v.	[255]

**Table 12:** Research progress of IL-12 in clinical trials.

Name	Tumor Type	ROA	Status	NCT Number
rhIL-12 and IL-2	Advanced Solid Tumors	i.v.+s.c.	Phase I	NCT00005604
recombinant IL-12	Primary Peritoneal Cavity Cancer Recurrent Ovarian Epithelial Cancer	i.p.	Phase II	NCT00016289
NHS-IL12	Malignant Epithelial Neoplasms, Malignant Epithelial Tumors, Malignant Mesenchymal Tumor	s.c.	Phase I	NCT01417546
NHS-IL12	Advanced HPV-Associated Malignancies	s.c.	Phase I/II	NCT04287868
NHS-IL12	Small Bowel and Colorectal Cancers	s.c.	Phase II	NCT04491955
NHS-IL12	Advanced Solid Tumors	i.v.	Phase Ib	NCT02994953
NHS-IL12	Kaposi’s Sarcoma	i.v.	Phase I/II	NCT04303117
NHS-IL12	Urothelial Cancer Bladder Cancer Genitourinary Cancer Urogenital Cancer	i.v.	Phase I	NCT04235777
NM-IL-12	Colostomy Stoma	s.c.	Phase IIa	NCT02544061
SON-1010 (IL12-FHAB)	Platinum-resistant Ovarian Cancer	/	Phase 1b/2a	NCT05756907
Ad5-yCD/mutTKSR39rep-hIL12	Prostate Cancer	i.t.	Phase I	NCT02555397
Adv/IL-12	Prostate Cancer	i.t.	Phase I	NCT00406939
Ad5-yCD/mutTKSR39rep-hIL12	Metastatic Pancreatic Cancer	i.t.	Phase I	NCT03281382
adenovirus-mediated human interleukin-12	Breast Cancer	i.t.	Phase I	NCT00849459
Ad.hIL-12	Radiorecurrent Prostate Cancer	i.p.	Phase I	NCT00110526
Ad-RTS-hIL-12	Melanoma	i.t.	Phase I/II	NCT01397708
Ad-RTS-hIL-12	Pediatric Brain Tumor Diffuse Intrinsic Pontine Glioma	i.t.	Phase I/II	NCT03330197
Ad-RTS-hIL-12	Glioblastoma Multiforme Anaplastic Oligoastrocytoma	i.t.	Phase I	NCT02026271
Ad-RTS-hIL-12	Glioblastoma	i.t.	Phase I	NCT03636477
Adv.RSV-hIL12	Breast Cancer Metastatic Cancer	i.t.	Phase I	NCT00301106
canarypox-hIL-12	Melanoma	i.t.	Phase I	NCT00003556
MEDI9253 (Recombinant Newcastle Disease Virus Encoding Interleukin-12)	Solid Tumors	i.t.	Phase I	NCT04613492
MEDI9253 + Durvalumab	Solid Tumors	i.t.	Phase I	NCT04613492
M032 (a Genetically Engineered HSV-1 Expressing IL-12)	Glioblastoma	i.t.	Phase I/II	NCT05084430
hTERT and IL-12 DNA	Breast Cancer Lung Cancer Pancreatic Cancer Head and Neck Cancer Ovarian Cancer ColoRectal Cancer Gastric Cancer Esophageal Cancer HepatoCellular Carcinoma	i.m.	Phase I	NCT02960594
IT-pIL12-EP	Triple-negative breast cancer	i.t.	Phase I	NCT02531425
IL-12p DNA	Malignant Melanoma	i.t.	Phase I	NCT00323206
IL-12 DNA	Metastatic Cancer	i.t.	Phase Ib	NCT00028652
Interleukin-12 cDNA	Colorectal Cancer Metastatic Cancer	i.t.	Phase I	NCT00072098
Interleukin-12 Plasmid	Merkel Cell Carcinoma	i.t.	Phase II	NCT01440816
INO-3112 (plasmid-encoding interleukin-12/HPV DNA plasmids) and durvalumab	Recurrent/Metastatic Human-Papilloma-Virus-Associated Cancers	i.m.	Phase II	NCT03439085
IMNN-001 (IL-12 Plasmid Formulated With PEG-PEI-Cholesterol Lipopolymer)	Epithelial Ovarian Cancer Fallopian Tube Cancer Primary Peritoneal Cancer	i.p.	Phase I	NCT02480374
Egen-001 (IL-12 Plasmid Formulated With PEG–PEI–Cholesterol Lipopolymer)	Ovarian Clear Cell Cystadenocarcinoma Ovarian Endometrioid Adenocarcinoma Ovarian Seromucinous Carcinoma	i.p.	Phase I	NCT01489371
EGEN-001 (IL-12 Plasmid Formulated With PEG–PEI–Cholesterol Lipopolymer)	Fallopian Tube Carcinoma Primary Peritoneal Carcinoma Recurrent Ovarian Carcinoma	i.p.	Phase II	NCT01118052
EGEN-001 and Pegylated Liposomal Doxorubicin Hydrochloride	Ovarian Clear Cell Cystadenocarcinoma Ovarian Endometrioid Adenocarcinoma Ovarian Seromucinous Carcinoma Ovarian Serous Cystadenocarcinoma Ovarian Undifferentiated Carcinoma Recurrent Fallopian Tube Carcinoma Recurrent Ovarian Carcinoma Recurrent Primary Peritoneal Carcinoma	i.p.	Phase I	NCT01489371
phIL12 GET	Basal Cell Carcinomas	i.t.	Phase I	NCT05077033
EGFR-IL12-CART	Metastatic Colorectal Cancer	/	Phase I/II	NCT03542799
Interleukin 12-Primed Activated T Cells (12ATC)	Melanoma	i.v.	Phase I	NCT00016055
Interleukin-12-Primed Activated T Cells (12ATC)	Colorectal Cancer Kidney Cancer	i.v.	Phase I	NCT00016042
Interleukin-12-Primed Activated T Cells in combination with 5FU, GM-CSF, and Interferon Alfa-2b	Colorectal Cancer Kidney Cancer	i.v.	Phase I/II	NCT00030342
EGFRt/19-28z/IL-12 CAR T Cells	Hematologic Malignancies	i.v.	Phase I	NCT06343376
CAR-T Cells (IL7 and CCL19 or/and IL12) Targeting Nectin4/FAP	Nectin4-positive Advanced Malignant Solid Tumor	i.t.	Phase I	NCT03932565
T-Cell Membrane-Anchored Tumor-Targeted Il12 (Attil12)	Soft Tissue Sarcoma Bone Sarcoma	i.v.	Phase 1	NCT05621668
IL-12 gene-transduced TIL	Melanoma	i.v.	Phase I/II	NCT01236573
Dendritic and Glioma Cells Fusion Vaccine With IL-12	Glioblastoma	i.d.	Phase I/II	NCT04388033
anti-ESO-1/IL-12 white blood cells	Metastatic Melanoma Metastatic Renal Cancer	i.v.	Phase I/II	NCT01457131
bacTRL-IL-12	Treatment-refractory Solid Tumors	i.v.	Phase I	NCT04025307

## References

[1] Flanigan R.C., Salmon S.E., Blumenstein B.A., Bearman S.I., Roy V., McGrath P.C., Caton J.R., Munshi N., Crawford E.D. (2001). Nephrectomy followed by interferon alfa-2b compared with interferon alfa-2b alone for metastatic renal-cell cancer. N. Engl. J. Med..

[2] Atkins M.B., Lotze M.T., Dutcher J.P., Fisher R.I., Weiss G., Margolin K., Abrams J., Sznol M., Parkinson D., Hawkins M. (1999). High-dose recombinant interleukin 2 therapy for patients with metastatic melanoma: Analysis of 270 patients treated between 1985 and 1993. J. Clin. Oncol..

[3] Del Vecchio M., Bajetta E., Canova S., Lotze M.T., Wesa A., Parmiani G., Anichini A. (2007). Interleukin-12: Biological properties and clinical application. Clin. Cancer Res..

[4] Weiss J.M., Subleski J.J., Wigginton J.M., Wiltrout R.H. (2007). Immunotherapy of cancer by IL-12-based cytokine combinations. Expert. Opin. Biol. Ther..

[5] Smyth M.J., Taniguchi M., Street S.E. (2000). The anti-tumor activity of IL-12: Mechanisms of innate immunity that are model and dose dependent. J. Immunol..

[6] Colombo M.P., Trinchieri G. (2002). Interleukin-12 in anti-tumor immunity and immunotherapy. Cytokine Growth Factor. Rev..

[7] Boggio K., Di Carlo E., Rovero S., Cavallo F., Quaglino E., Lollini P.L., Nanni P., Nicoletti G., Wolf S., Musiani P. (2000). Ability of systemic interleukin-12 to hamper progressive stages of mammary carcinogenesis in HER2/neu transgenic mice. Cancer Res..

[8] Nguyen K.G., Vrabel M.R., Mantooth S.M., Hopkins J.J., Wagner E.S., Gabaldon T.A., Zaharoff D.A. (2020). Localized Interleukin-12 for Cancer Immunotherapy. Front. Immunol..

[9] Chiocca E.A., Gelb A.B., Chen C.C., Rao G., Reardon D.A., Wen P.Y., Bi W.L., Peruzzi P., Amidei C., Triggs D. (2022). Combined immunotherapy with controlled interleukin-12 gene therapy and immune checkpoint blockade in recurrent glioblastoma: An open-label, multi-institutional phase I trial. Neuro Oncol..

[10] Mirlekar B., Pylayeva-Gupta Y. (2021). IL-12 Family Cytokines in Cancer and Immunotherapy. Cancers.

[11] Cohen J. (1995). IL-12 deaths: Explanation and a puzzle. Science.

[12] Leonard J.P., Sherman M.L., Fisher G.L., Buchanan L.J., Larsen G., Atkins M.B., Sosman J.A., Dutcher J.P., Vogelzang N.J., Ryan J.L. (1997). Effects of single-dose interleukin-12 exposure on interleukin-12-associated toxicity and interferon-gamma production. Blood.

[13] Gollob J.A., Mier J.W., Veenstra K., McDermott D.F., Clancy D., Clancy M., Atkins M.B. (2000). Phase I trial of twice-weekly intravenous interleukin 12 in patients with metastatic renal cell cancer or malignant melanoma: Ability to maintain IFN-gamma induction is associated with clinical response. Clin. Cancer Res..

[14] Schilbach K., Alkhaled M., Welker C., Eckert F., Blank G., Ziegler H., Sterk M., Müller F., Sonntag K., Wieder T. (2015). Cancer-targeted IL-12 controls human rhabdomyosarcoma by senescence induction and myogenic differentiation. Oncoimmunology.

[15] Salem M.L., Gillanders W.E., Kadima A.N., El-Naggar S., Rubinstein M.P., Demcheva M., Vournakis J.N., Cole D.J. (2006). Review: Novel nonviral delivery approaches for interleukin-12 protein and gene systems: Curbing toxicity and enhancing adjuvant activity. J. Interferon Cytokine Res..

[16] Sangro B., Melero I., Qian C., Prieto J. (2005). Gene therapy of cancer based on interleukin 12. Curr. Gene Ther..

[17] Xue D., Moon B., Liao J., Guo J., Zou Z., Han Y., Cao S., Wang Y., Fu Y.X., Peng H. (2022). A tumor-specific pro-IL-12 activates preexisting cytotoxic T cells to control established tumors. Sci. Immunol..

[18] Mansurov A., Hosseinchi P., Chang K., Lauterbach A.L., Gray L.T., Alpar A.T., Budina E., Slezak A.J., Kang S., Cao S. (2022). Masking the immunotoxicity of interleukin-12 by fusing it with a domain of its receptor via a tumour-protease-cleavable linker. Nat. Biomed. Eng..

[19] Momin N., Mehta N.K., Bennett N.R., Ma L., Palmeri J.R., Chinn M.M., Lutz E.A., Kang B., Irvine D.J., Spranger S. (2019). Anchoring of intratumorally administered cytokines to collagen safely potentiates systemic cancer immunotherapy. Sci. Transl. Med..

[20] Mansurov A., Ishihara J., Hosseinchi P., Potin L., Marchell T.M., Ishihara A., Williford J.M., Alpar A.T., Raczy M.M., Gray L.T. (2020). Collagen-binding IL-12 enhances tumour inflammation and drives the complete remission of established immunologically cold mouse tumours. Nat. Biomed. Eng..

[21] Ongaro T., Matasci M., Cazzamalli S., Gouyou B., De Luca R., Neri D., Villa A. (2019). A novel anti-cancer L19-interleukin-12 fusion protein with an optimized peptide linker efficiently localizes in vivo at the site of tumors. J. Biotechnol..

[22] Gafner V., Trachsel E., Neri D. (2006). An engineered antibody-interleukin-12 fusion protein with enhanced tumor vascular targeting properties. Int. J. Cancer.

[23] Fallon J.K., Vandeveer A.J., Schlom J., Greiner J.W. (2017). Enhanced antitumor effects by combining an IL-12/anti-DNA fusion protein with avelumab, an anti-PD-L1 antibody. Oncotarget.

[24] Xu C., Marelli B., Qi J., Qin G., Yu H., Wang H., Jenkins M.H., Lo K.-M., Lan Y. (2022). NHS-IL12 and bintrafusp alfa combination therapy enhances antitumor activity in preclinical cancer models. Transl. Oncol..

[25] Xu C., Zhang Y., Rolfe P.A., Hernández V.M., Guzman W., Kradjian G., Marelli B., Qin G., Qi J., Wang H. (2017). Combination Therapy with NHS-muIL12 and Avelumab (anti-PD-L1) Enhances Antitumor Efficacy in Preclinical Cancer Models. Clin. Cancer Res..

[26] Cini J.K., Dexter S., Rezac D.J., McAndrew S.J., Hedou G., Brody R., Eraslan R.N., Kenney R.T., Mohan P. (2023). SON-1210—A novel bifunctional IL-12/IL-15 fusion protein that improves cytokine half-life, targets tumors, and enhances therapeutic efficacy. Front. Immunol..

[27] Pan W.Y., Lo C.H., Chen C.C., Wu P.Y., Roffler S.R., Shyue S.K., Tao M.H. (2012). Cancer immunotherapy using a membrane-bound interleukin-12 with B7-1 transmembrane and cytoplasmic domains. Mol. Ther..

[28] Nasu Y., Bangma C.H., Hull G.W., Lee H.M., Hu J., Wang J., McCurdy M.A., Shimura S., Yang G., Timme T.L. (1999). Adenovirus-mediated interleukin-12 gene therapy for prostate cancer: Suppression of orthotopic tumor growth and pre-established lung metastases in an orthotopic model. Gene Ther..

[29] Martuza R.L., Malick A., Markert J.M., Ruffner K.L., Coen D.M. (1991). Experimental therapy of human glioma by means of a genetically engineered virus mutant. Science.

[30] Macedo N., Miller D.M., Haq R., Kaufman H.L. (2020). Clinical landscape of oncolytic virus research in 2020. J. Immunother Cancer.

[31] Sheridan C. (2015). First oncolytic virus edges towards approval in surprise vote. Nat. Biotechnol..

[32] Toda M., Martuza R.L., Rabkin S.D. (2001). Combination suicide/cytokine gene therapy as adjuvants to a defective herpes simplex virus-based cancer vaccine. Gene Ther..

[33] Chouljenko D.V., Ding J., Lee I.F., Murad Y.M., Bu X., Liu G., Delwar Z., Sun Y., Yu S., Samudio I. (2020). Induction of Durable Antitumor Response by a Novel Oncolytic Herpesvirus Expressing Multiple Immunomodulatory Transgenes. Biomedicines.

[34] Hu H., Zhang S., Cai L., Duan H., Li Y., Yang J., Wang Y., Liu B., Dong S., Fang Z. (2022). A novel cocktail therapy based on quintuplet combination of oncolytic herpes simplex virus-2 vectors armed with interleukin-12, interleukin-15, GM-CSF, PD1v, and IL-7 × CCL19 results in enhanced antitumor efficacy. Virol. J..

[35] Bennett J.J., Malhotra S., Wong R.J., Delman K., Zager J., St-Louis M., Johnson P., Fong Y. (2001). Interleukin 12 secretion enhances antitumor efficacy of oncolytic herpes simplex viral therapy for colorectal cancer. Ann. Surg..

[36] Zhang N., Li J., Yu J., Wan Y., Zhang C., Zhang H., Cao Y. (2023). Construction of an IL12 and CXCL11 armed oncolytic herpes simplex virus using the CRISPR/Cas9 system for colon cancer treatment. Virus Res..

[37] Ghouse S.M., Nguyen H.M., Bommareddy P.K., Guz-Montgomery K., Saha D. (2020). Oncolytic Herpes Simplex Virus Encoding IL12 Controls Triple-Negative Breast Cancer Growth and Metastasis. Front. Oncol..

[38] Saha D., Wakimoto H., Peters C.W., Antoszczyk S.J., Rabkin S.D., Martuza R.L. (2018). Combinatorial Effects of VEGFR Kinase Inhibitor Axitinib and Oncolytic Virotherapy in Mouse and Human Glioblastoma Stem-Like Cell Models. Clin. Cancer Res..

[39] Saha D., Rabkin S.D., Martuza R.L. (2020). Temozolomide antagonizes oncolytic immunovirotherapy in glioblastoma. J. Immunother. Cancer.

[40] Saha D., Martuza R.L., Rabkin S.D. (2018). Oncolytic herpes simplex virus immunovirotherapy in combination with immune checkpoint blockade to treat glioblastoma. Immunotherapy.

[41] Saha D., Martuza R.L., Rabkin S.D. (2017). Macrophage Polarization Contributes to Glioblastoma Eradication by Combination Immunovirotherapy and Immune Checkpoint Blockade. Cancer Cell.

[42] Ino Y., Saeki Y., Fukuhara H., Todo T. (2006). Triple combination of oncolytic herpes simplex virus-1 vectors armed with interleukin-12, interleukin-18, or soluble B7-1 results in enhanced antitumor efficacy. Clin. Cancer Res..

[43] Zhang W., Fulci G., Wakimoto H., Cheema T.A., Buhrman J.S., Jeyaretna D.S., Stemmer Rachamimov A.O., Rabkin S.D., Martuza R.L. (2013). Combination of oncolytic herpes simplex viruses armed with angiostatin and IL-12 enhances antitumor efficacy in human glioblastoma models. Neoplasia.

[44] Wang L., Zhou X., Chen X., Liu Y., Huang Y., Cheng Y., Ren P., Zhao J., Zhou G.G. (2024). Enhanced therapeutic efficacy for glioblastoma immunotherapy with an oncolytic herpes simplex virus armed with anti-PD-1 antibody and IL-12. Mol. Ther. Oncol..

[45] Bommareddy P.K., Wakimoto H., Martuza R.L., Kaufman H.L., Rabkin S.D., Saha D. (2024). Oncolytic herpes simplex virus expressing IL-2 controls glioblastoma growth and improves survival. J. Immunother. Cancer.

[46] De Lucia M., Cotugno G., Bignone V., Garzia I., Nocchi L., Langone F., Petrovic B., Sasso E., Pepe S., Froechlich G. (2020). Retargeted and Multi-cytokine-Armed Herpes Virus Is a Potent Cancer Endovaccine for Local and Systemic Anti-tumor Treatment. Mol. Ther. Oncolytics.

[47] Chouljenko D.V., Murad Y.M., Lee I.F., Delwar Z., Ding J., Liu G., Liu X., Bu X., Sun Y., Samudio I. (2023). Targeting carcinoembryonic antigen-expressing tumors using a novel transcriptional and translational dual-regulated oncolytic herpes simplex virus type 1. Mol. Ther. Oncolytics.

[48] Kim K.J., Moon D., Kong S.J., Lee Y.S., Yoo Y., Kim S., Kim C., Chon H.J., Kim J.H., Choi K.J. (2021). Antitumor effects of IL-12 and GM-CSF co-expressed in an engineered oncolytic HSV-1. Gene Ther..

[49] Todo T., Martuza R.L., Rabkin S.D., Johnson P.A. (2001). Oncolytic herpes simplex virus vector with enhanced MHC class I presentation and tumor cell killing. Proc. Natl. Acad. Sci. USA.

[50] Antoszczyk S., Spyra M., Mautner V.F., Kurtz A., Stemmer-Rachamimov A.O., Martuza R.L., Rabkin S.D. (2014). Treatment of orthotopic malignant peripheral nerve sheath tumors with oncolytic herpes simplex virus. Neuro Oncol..

[51] Cheema T.A., Wakimoto H., Fecci P.E., Ning J., Kuroda T., Jeyaretna D.S., Martuza R.L., Rabkin S.D. (2013). Multifaceted oncolytic virus therapy for glioblastoma in an immunocompetent cancer stem cell model. Proc. Natl. Acad. Sci. USA.

[52] Wong R.J., Chan M.K., Yu Z., Kim T.H., Bhargava A., Stiles B.M., Horsburgh B.C., Shah J.P., Ghossein R.A., Singh B. (2004). Effective intravenous therapy of murine pulmonary metastases with an oncolytic herpes virus expressing interleukin 12. Clin. Cancer Res..

[53] Passer B.J., Cheema T., Wu S., Wu C.L., Rabkin S.D., Martuza R.L. (2013). Combination of vinblastine and oncolytic herpes simplex virus vector expressing IL-12 therapy increases antitumor and antiangiogenic effects in prostate cancer models. Cancer Gene Ther..

[54] Wong R.J., Chan M.K., Yu Z., Ghossein R.A., Ngai I., Adusumilli P.S., Stiles B.M., Shah J.P., Singh B., Fong Y. (2004). Angiogenesis inhibition by an oncolytic herpes virus expressing interleukin 12. Clin. Cancer Res..

[55] Wong R.J., Patel S.G., Kim S., DeMatteo R.P., Malhotra S., Bennett J.J., St-Louis M., Shah J.P., Johnson P.A., Fong Y. (2001). Cytokine gene transfer enhances herpes oncolytic therapy in murine squamous cell carcinoma. Hum. Gene Ther..

[56] Varghese S., Rabkin S.D., Liu R., Nielsen P.G., Ipe T., Martuza R.L. (2006). Enhanced therapeutic efficacy of IL-12, but not GM-CSF, expressing oncolytic herpes simplex virus for transgenic mouse derived prostate cancers. Cancer Gene Ther..

[57] Jarnagin W.R., Zager J.S., Klimstra D., Delman K.A., Malhotra S., Ebright M., Little S., DeRubertis B., Stanziale S.F., Hezel M. (2003). Neoadjuvant treatment of hepatic malignancy: An oncolytic herpes simplex virus expressing IL-12 effectively treats the parent tumor and protects against recurrence-after resection. Cancer Gene Ther..

[58] Parker J.N., Meleth S., Hughes K.B., Gillespie G.Y., Whitley R.J., Markert J.M. (2005). Enhanced inhibition of syngeneic murine tumors by combinatorial therapy with genetically engineered HSV-1 expressing CCL2 and IL-12. Cancer Gene Ther..

[59] Ring E.K., Li R., Moore B.P., Nan L., Kelly V.M., Han X., Beierle E.A., Markert J.M., Leavenworth J.W., Gillespie G.Y. (2017). Newly Characterized Murine Undifferentiated Sarcoma Models Sensitive to Virotherapy with Oncolytic HSV-1 M002. Mol. Ther. Oncolytics.

[60] Friedman G.K., Bernstock J.D., Chen D., Nan L., Moore B.P., Kelly V.M., Youngblood S.L., Langford C.P., Han X., Ring E.K. (2018). Enhanced Sensitivity of Patient-Derived Pediatric High-Grade Brain Tumor Xenografts to Oncolytic HSV-1 Virotherapy Correlates with Nectin-1 Expression. Sci. Rep..

[61] Cody J.J., Scaturro P., Cantor A.B., Yancey Gillespie G., Parker J.N., Markert J.M. (2012). Preclinical evaluation of oncolytic δγ(1)34.5 herpes simplex virus expressing interleukin-12 for therapy of breast cancer brain metastases. Int. J. Breast Cancer.

[62] Gillory L.A., Megison M.L., Stewart J.E., Mroczek-Musulman E., Nabers H.C., Waters A.M., Kelly V., Coleman J.M., Markert J.M., Gillespie G.Y. (2013). Preclinical evaluation of engineered oncolytic herpes simplex virus for the treatment of neuroblastoma. PLoS ONE.

[63] Megison M.L., Gillory L.A., Stewart J.E., Nabers H.C., Mroczek-Musulman E., Waters A.M., Coleman J.M., Kelly V., Markert J.M., Gillespie G.Y. (2014). Preclinical evaluation of engineered oncolytic herpes simplex virus for the treatment of pediatric solid tumors. PLoS ONE.

[64] Leoni V., Vannini A., Gatta V., Rambaldi J., Sanapo M., Barboni C., Zaghini A., Nanni P., Lollini P.L., Casiraghi C. (2018). A fully-virulent retargeted oncolytic HSV armed with IL-12 elicits local immunity and vaccine therapy towards distant tumors. PLoS Pathog..

[65] Alessandrini F., Menotti L., Avitabile E., Appolloni I., Ceresa D., Marubbi D., Campadelli-Fiume G., Malatesta P. (2019). Eradication of glioblastoma by immuno-virotherapy with a retargeted oncolytic HSV in a preclinical model. Oncogene.

[66] Yan R., Zhou X., Chen X., Liu X., Tang Y., Ma J., Zhou G.G., ImmVira Group Corporation (2019). Enhancement of Oncolytic Activity of oHSV Expressing IL-12 and Anti PD-1 Antibody by Concurrent Administration of Exosomes Carrying CTLA-4 miRNA. Immunotherapy.

[67] Xiao X., Li J., McCown T.J., Samulski R.J. (1997). Gene transfer by adeno-associated virus vectors into the central nervous system. Exp. Neurol..

[68] Xiao X., Li J., Samulski R.J. (1998). Production of high-titer recombinant adeno-associated virus vectors in the absence of helper adenovirus. J. Virol..

[69] Xiao X., Li J., Samulski R.J. (1996). Efficient long-term gene transfer into muscle tissue of immunocompetent mice by adeno-associated virus vector. J. Virol..

[70] Vanrell L., Di Scala M., Blanco L., Otano I., Gil-Farina I., Baldim V., Paneda A., Berraondo P., Beattie S.G., Chtarto A. (2011). Development of a liver-specific Tet-on inducible system for AAV vectors and its application in the treatment of liver cancer. Mol. Ther..

[71] Daya S., Berns K.I. (2008). Gene therapy using adeno-associated virus vectors. Clin. Microbiol. Rev..

[72] Kanagawa N., Gao J.Q., Motomura Y., Yanagawa T., Mukai Y., Yoshioka Y., Okada N., Nakagawa S. (2008). Antitumor mechanism of intratumoral injection with IL-12-expressing adenoviral vector against IL-12-unresponsive tumor. Biochem. Biophys. Res. Commun..

[73] Thaci B., Ahmed A.U., Ulasov I.V., Wainwright D.A., Nigam P., Auffinger B., Tobias A.L., Han Y., Zhang L., Moon K.S. (2014). Depletion of myeloid-derived suppressor cells during interleukin-12 immunogene therapy does not confer a survival advantage in experimental malignant glioma. Cancer Gene Ther..

[74] Chiu T.L., Lin S.Z., Hsieh W.H., Peng C.W. (2009). AAV2-mediated interleukin-12 in the treatment of malignant brain tumors through activation of NK cells. Int. J. Oncol..

[75] Chiu T.L., Wang M.J., Su C.C. (2012). The treatment of glioblastoma multiforme through activation of microglia and TRAIL induced by rAAV2-mediated IL-12 in a syngeneic rat model. J. Biomed. Sci..

[76] Mazzolini G., Qian C., Xie X., Sun Y., Lasarte J.J., Drozdzik M., Prieto J. (1999). Regression of colon cancer and induction of antitumor immunity by intratumoral injection of adenovirus expressing interleukin-12. Cancer Gene Ther..

[77] Caruso M., Pham-Nguyen K., Kwong Y.L., Xu B., Kosai K.I., Finegold M., Woo S.L., Chen S.H. (1996). Adenovirus-mediated interleukin-12 gene therapy for metastatic colon carcinoma. Proc. Natl. Acad. Sci. USA.

[78] Huang J.H., Zhang S.N., Choi K.J., Choi I.K., Kim J.H., Lee M.G., Lee M., Kim H., Yun C.O. (2010). Therapeutic and tumor-specific immunity induced by combination of dendritic cells and oncolytic adenovirus expressing IL-12 and 4-1BBL. Mol. Ther..

[79] Zhang S.N., Choi I.K., Huang J.H., Yoo J.Y., Choi K.J., Yun C.O. (2011). Optimizing DC vaccination by combination with oncolytic adenovirus coexpressing IL-12 and GM-CSF. Mol. Ther..

[80] Lo C.H., Chang C.M., Tang S.W., Pan W.Y., Fang C.C., Chen Y., Wu P.Y., Chen K.Y., Ma H.I., Xiao X. (2010). Differential antitumor effect of interleukin-12 family cytokines on orthotopic hepatocellular carcinoma. J. Gene Med..

[81] Schmitz V., Tirado-Ledo L., Raskopf E., Rabe C., Wernert N., Wang L., Prieto J., Qian C., Sauerbruch T., Caselmann W.H. (2005). Effective antitumour mono- and combination therapy by gene delivery of angiostatin-like molecule and interleukin-12 in a murine hepatoma model. Int. J. Colorectal Dis..

[82] Fujita T., Timme T.L., Tabata K., Naruishi K., Kusaka N., Watanabe M., Abdelfattah E., Zhu J.X., Ren C., Ren C. (2007). Cooperative effects of adenoviral vector-mediated interleukin 12 gene therapy with radiotherapy in a preclinical model of metastatic prostate cancer. Gene Ther..

[83] Gonzalez-Aparicio M., Alzuguren P., Mauleon I., Medina-Echeverz J., Hervas-Stubbs S., Mancheno U., Berraondo P., Crettaz J., Gonzalez-Aseguinolaza G., Prieto J. (2011). Oxaliplatin in combination with liver-specific expression of interleukin 12 reduces the immunosuppressive microenvironment of tumours and eradicates metastatic colorectal cancer in mice. Gut.

[84] Oh E., Oh J.E., Hong J., Chung Y., Lee Y., Park K.D., Kim S., Yun C.O. (2017). Optimized biodegradable polymeric reservoir-mediated local and sustained co-delivery of dendritic cells and oncolytic adenovirus co-expressing IL-12 and GM-CSF for cancer immunotherapy. J. Control. Release.

[85] Hwang K.S., Cho W.K., Yoo J., Yun H.J., Kim S., Im D.S. (2005). Adenovirus-mediated interleukin-12 gene transfer combined with cytosine deaminase followed by 5-fluorocytosine treatment exerts potent antitumor activity in Renca tumor-bearing mice. BMC Cancer.

[86] Düchs M.J., Kratzer R.F., Vieyra-Garcia P., Strobel B., Schönberger T., Groß P., Aljayyoussi G., Gupta A., Lang I., Klein H. (2024). Riboswitch-controlled IL-12 gene therapy reduces hepatocellular cancer in mice. Front. Immunol..

[87] Barrett J.A., Cai H., Miao J., Khare P.D., Gonzalez P., Dalsing-Hernandez J., Sharma G., Chan T., Cooper L.J.N., Lebel F. (2018). Regulated intratumoral expression of IL-12 using a RheoSwitch Therapeutic System(^®^) (RTS(^®^)) gene switch as gene therapy for the treatment of glioma. Cancer Gene Ther..

[88] Choi K.J., Zhang S.N., Choi I.K., Kim J.S., Yun C.O. (2012). Strengthening of antitumor immune memory and prevention of thymic atrophy mediated by adenovirus expressing IL-12 and GM-CSF. Gene Ther..

[89] Kirchhammer N., Trefny M.P., Natoli M., Brücher D., Smith S.N., Werner F., Koch V., Schreiner D., Bartoszek E., Buchi M. (2022). NK cells with tissue-resident traits shape response to immunotherapy by inducing adaptive antitumor immunity. Sci. Transl. Med..

[90] Poutou J., Bunuales M., Gonzalez-Aparicio M., Garcia-Aragoncillo E., Quetglas J.I., Casado R., Bravo-Perez C., Alzuguren P., Hernandez-Alcoceba R. (2015). Safety and antitumor effect of oncolytic and helper-dependent adenoviruses expressing interleukin-12 variants in a hamster pancreatic cancer model. Gene Ther..

[91] Bortolanza S., Bunuales M., Otano I., Gonzalez-Aseguinolaza G., Ortiz-de-Solorzano C., Perez D., Prieto J., Hernandez-Alcoceba R. (2009). Treatment of pancreatic cancer with an oncolytic adenovirus expressing interleukin-12 in Syrian hamsters. Mol. Ther..

[92] Sangro B., Mazzolini G., Ruiz J., Herraiz M., Quiroga J., Herrero I., Benito A., Larrache J., Pueyo J., Subtil J.C. (2004). Phase I trial of intratumoral injection of an adenovirus encoding interleukin-12 for advanced digestive tumors. J. Clin. Oncol..

[93] Choi I.K., Lee J.S., Zhang S.N., Park J., Sonn C.H., Lee K.M., Yun C.O. (2011). Oncolytic adenovirus co-expressing IL-12 and IL-18 improves tumor-specific immunity via differentiation of T cells expressing IL-12Rβ2 or IL-18Rα. Gene Ther..

[94] Lee Y.S., Kim J.H., Choi K.J., Choi I.K., Kim H., Cho S., Cho B.C., Yun C.O. (2006). Enhanced antitumor effect of oncolytic adenovirus expressing interleukin-12 and B7-1 in an immunocompetent murine model. Clin. Cancer Res..

[95] Narvaiza I., Mazzolini G., Barajas M., Duarte M., Zaratiegui M., Qian C., Melero I., Prieto J. (2000). Intratumoral coinjection of two adenoviruses, one encoding the chemokine IFN-gamma-inducible protein-10 and another encoding IL-12, results in marked antitumoral synergy. J. Immunol..

[96] Gyorffy S., Palmer K., Podor T.J., Hitt M., Gauldie J. (2001). Combined treatment of a murine breast cancer model with type 5 adenovirus vectors expressing murine angiostatin and IL-12: A role for combined anti-angiogenesis and immunotherapy. J. Immunol..

[97] Hall S.J., Canfield S.E., Yan Y., Hassen W., Selleck W.A., Chen S.H. (2002). A novel bystander effect involving tumor cell-derived Fas and FasL interactions following Ad.HSV-tk and Ad.mIL-12 gene therapies in experimental prostate cancer. Gene Ther..

[98] Wang L., Hernández-Alcoceba R., Shankar V., Zabala M., Kochanek S., Sangro B., Kramer M.G., Prieto J., Qian C. (2004). Prolonged and inducible transgene expression in the liver using gutless adenovirus: A potential therapy for liver cancer. Gastroenterology.

[99] Chang C.J., Chen Y.H., Huang K.W., Cheng H.W., Chan S.F., Tai K.F., Hwang L.H. (2007). Combined GM-CSF and IL-12 gene therapy synergistically suppresses the growth of orthotopic liver tumors. Hepatology.

[100] Chen S.H., Pham-Nguyen K.B., Martinet O., Huang Y., Yang W., Thung S.N., Chen L., Mittler R., Woo S.L. (2000). Rejection of disseminated metastases of colon carcinoma by synergism of IL-12 gene therapy and 4-1BB costimulation. Mol. Ther..

[101] Fang L., Tian W., Zhang C., Wang X., Li W., Zhang Q., Zhang Y., Zheng J. (2023). Oncolytic adenovirus-mediated expression of CCL5 and IL12 facilitates CA9-targeting CAR-T therapy against renal cell carcinoma. Pharmacol. Res..

[102] Mercer J., Helenius A. (2008). Vaccinia virus uses macropinocytosis and apoptotic mimicry to enter host cells. Science.

[103] Hiley C.T., Yuan M., Lemoine N.R., Wang Y. (2010). Lister strain vaccinia virus, a potential therapeutic vector targeting hypoxic tumours. Gene Ther..

[104] Lu S., Zhang Z., Du P., Chard L.S., Yan W., El Khouri M., Wang Z., Zhang Z., Chu Y., Gao D. (2020). A Virus-Infected, Reprogrammed Somatic Cell-Derived Tumor Cell (VIReST) Vaccination Regime Can Prevent Initiation and Progression of Pancreatic Cancer. Clin. Cancer Res..

[105] Hou W., Chen H., Rojas J., Sampath P., Thorne S.H. (2014). Oncolytic vaccinia virus demonstrates antiangiogenic effects mediated by targeting of VEGF. Int. J. Cancer.

[106] Breitbach C.J., Burke J., Jonker D., Stephenson J., Haas A.R., Chow L.Q., Nieva J., Hwang T.H., Moon A., Patt R. (2011). Intravenous delivery of a multi-mechanistic cancer-targeted oncolytic poxvirus in humans. Nature.

[107] Nakao S., Arai Y., Tasaki M., Yamashita M., Murakami R., Kawase T., Amino N., Nakatake M., Kurosaki H., Mori M. (2020). Intratumoral expression of IL-7 and IL-12 using an oncolytic virus increases systemic sensitivity to immune checkpoint blockade. Sci. Transl. Med..

[108] Chen B., Timiryasova T.M., Haghighat P., Andres M.L., Kajioka E.H., Dutta-Roy R., Gridley D.S., Fodor I. (2001). Low-dose vaccinia virus-mediated cytokine gene therapy of glioma. J. Immunother..

[109] Chen B., Timiryasova T.M., Andres M.L., Kajioka E.H., Dutta-Roy R., Gridley D.S., Fodor I. (2000). Evaluation of combined vaccinia virus-mediated antitumor gene therapy with p53, IL-2, and IL-12 in a glioma model. Cancer Gene Ther..

[110] Ahmed J., Chard L.S., Yuan M., Wang J., Howells A., Li Y., Li H., Zhang Z., Lu S., Gao D. (2020). A new oncolytic Vacciniavirus augments antitumor immune responses to prevent tumor recurrence and metastasis after surgery. J. Immunother. Cancer.

[111] Kaufman H.L., Flanagan K., Lee C.S., Perretta D.J., Horig H. (2002). Insertion of interleukin-2 (IL-2) and interleukin-12 (IL-12) genes into vaccinia virus results in effective anti-tumor responses without toxicity. Vaccine.

[112] Jackaman C., Nelson D.J. (2010). Cytokine-armed vaccinia virus infects the mesothelioma tumor microenvironment to overcome immune tolerance and mediate tumor resolution. Cancer Gene Ther..

[113] Martin N.T., Crupi M.J.F., Taha Z., Poutou J., Whelan J.T., Vallati S., Petryk J., Marius R., Austin B., Azad T. (2023). Engineering Rapalog-Inducible Genetic Switches Based on Split-T7 Polymerase to Regulate Oncolytic Virus-Driven Production of Tumour-Localized IL-12 for Anti-Cancer Immunotherapy. Pharmaceuticals.

[114] Ge Y., Wang H., Ren J., Liu W., Chen L., Chen H., Ye J., Dai E., Ma C., Ju S. (2020). Oncolytic vaccinia virus delivering tethered IL-12 enhances antitumor effects with improved safety. J. Immunother. Cancer.

[115] Kurokawa C., Agrawal S., Mitra A., Galvani E., Burke S., Varshine A., Rothstein R., Schifferli K., Monks N.R., Foloppe J. (2024). Mediation of antitumor activity by AZD4820 oncolytic vaccinia virus encoding IL-12. Mol. Ther. Oncol..

[116] Seclì L., Infante L., Nocchi L., De Lucia M., Cotugno G., Leoni G., Micarelli E., Garzia I., Avalle L., Sdruscia G. (2023). Vector Aided Microenvironment programming (VAMP): Reprogramming the TME with MVA virus expressing IL-12 for effective antitumor activity. J. Immunother. Cancer.

[117] Bella Á., Arrizabalaga L., Di Trani C.A., Gonzalez-Gomariz J., Gomar C., Russo-Cabrera J.S., Olivera I., Cirella A., Fernandez-Sendin M., Alvarez M. (2023). Intraperitoneal administration of a modified vaccinia virus Ankara confers single-chain interleukin-12 expression to the omentum and achieves immune-mediated efficacy against peritoneal carcinomatosis. J. Immunother. Cancer.

[118] Backhaus P.S., Veinalde R., Hartmann L., Dunder J.E., Jeworowski L.M., Albert J., Hoyler B., Poth T., Jäger D., Ungerechts G. (2019). Immunological Effects and Viral Gene Expression Determine the Efficacy of Oncolytic Measles Vaccines Encoding IL-12 or IL-15 Agonists. Viruses.

[119] Veinalde R., Grossardt C., Hartmann L., Bourgeois-Daigneault M.C., Bell J.C., Jäger D., von Kalle C., Ungerechts G., Engeland C.E. (2017). Oncolytic measles virus encoding interleukin-12 mediates potent antitumor effects through T cell activation. Oncoimmunology.

[120] Najmuddin S., Amin Z.M., Tan S.W., Yeap S.K., Kalyanasundram J., Ani M.A.C., Veerakumarasivam A., Chan S.C., Chia S.L., Yusoff K. (2020). Cytotoxicity study of the interleukin-12-expressing recombinant Newcastle disease virus strain, rAF-IL12, towards CT26 colon cancer cells in vitro and in vivo. Cancer Cell Int..

[121] Ren G., Tian G., Liu Y., He J., Gao X., Yu Y., Liu X., Zhang X., Sun T., Liu S. (2016). Recombinant Newcastle Disease Virus Encoding IL-12 and/or IL-2 as Potential Candidate for Hepatoma Carcinoma Therapy. Technol. Cancer Res. Treat..

[122] Syed Najmuddin S.U.F., Amin Z.M., Tan S.W., Yeap S.K., Kalyanasundram J., Veerakumarasivam A., Chan S.C., Chia S.L., Yusoff K., Alitheen N.B. (2020). Oncolytic effects of the recombinant Newcastle disease virus, rAF-IL12, against colon cancer cells in vitro and in tumor-challenged NCr-Foxn1nu nude mice. PeerJ.

[123] Asselin-Paturel C., Lassau N., Guinebretière J.M., Zhang J., Gay F., Bex F., Hallez S., Leclere J., Peronneau P., Mami-Chouaib F. (1999). Transfer of the murine interleukin-12 gene in vivo by a Semliki Forest virus vector induces B16 tumor regression through inhibition of tumor blood vessel formation monitored by Doppler ultrasonography. Gene Ther..

[124] Colmenero P., Chen M., Castaños-Velez E., Liljeström P., Jondal M. (2002). Immunotherapy with recombinant SFV-replicons expressing the P815A tumor antigen or IL-12 induces tumor regression. Int. J. Cancer.

[125] Melero I., Quetglas J.I., Reboredo M., Dubrot J., Rodriguez-Madoz J.R., Mancheño U., Casales E., Riezu-Boj J.I., Ruiz-Guillen M., Ochoa M.C. (2015). Strict requirement for vector-induced type I interferon in efficacious antitumor responses to virally encoded IL12. Cancer Res..

[126] Roche F.P., Sheahan B.J., O’Mara S.M., Atkins G.J. (2010). Semliki Forest virus-mediated gene therapy of the RG2 rat glioma. Neuropathol. Appl. Neurobiol..

[127] Quetglas J.I., Labiano S., Aznar M., Bolaños E., Azpilikueta A., Rodriguez I., Casales E., Sánchez-Paulete A.R., Segura V., Smerdou C. (2015). Virotherapy with a Semliki Forest Virus-Based Vector Encoding IL12 Synergizes with PD-1/PD-L1 Blockade. Cancer Immunol. Res..

[128] Quetglas J.I., Dubrot J., Bezunartea J., Sanmamed M.F., Hervas-Stubbs S., Smerdou C., Melero I. (2012). Immunotherapeutic synergy between anti-CD137 mAb and intratumoral administration of a cytopathic Semliki Forest virus encoding IL-12. Mol. Ther..

[129] Yamanaka R., Zullo S.A., Tanaka R., Ramsey J., Blaese M., Xanthopoulos K.G. (2000). Induction of a therapeutic antitumor immunological response by intratumoral injection of genetically engineered Semliki Forest virus to produce interleukin-12. Neurosurg. Focus.

[130] Chikkanna-Gowda C.P., Sheahan B.J., Fleeton M.N., Atkins G.J. (2005). Regression of mouse tumours and inhibition of metastases following administration of a Semliki Forest virus vector with enhanced expression of IL-12. Gene Ther..

[131] Rodriguez-Madoz J.R., Prieto J., Smerdou C. (2005). Semliki forest virus vectors engineered to express higher IL-12 levels induce efficient elimination of murine colon adenocarcinomas. Mol. Ther..

[132] Quetglas J.I., Rodriguez-Madoz J.R., Bezunartea J., Ruiz-Guillen M., Casales E., Medina-Echeverz J., Prieto J., Berraondo P., Hervas-Stubbs S., Smerdou C. (2013). Eradication of liver-implanted tumors by Semliki Forest virus expressing IL-12 requires efficient long-term immune responses. J. Immunol..

[133] Rodriguez-Madoz J.R., Zabala M., Alfaro M., Prieto J., Kramer M.G., Smerdou C. (2014). Short-term intratumoral interleukin-12 expressed from an alphaviral vector is sufficient to induce an efficient antitumoral response against spontaneous hepatocellular carcinomas. Hum. Gene Ther..

[134] Rodriguez-Madoz J.R., Liu K.H., Quetglas J.I., Ruiz-Guillen M., Otano I., Crettaz J., Butler S.D., Bellezza C.A., Dykes N.L., Tennant B.C. (2009). Semliki forest virus expressing interleukin-12 induces antiviral and antitumoral responses in woodchucks with chronic viral hepatitis and hepatocellular carcinoma. J. Virol..

[135] Ren H., Boulikas T., Lundstrom K., Söling A., Warnke P.C., Rainov N.G. (2003). Immunogene therapy of recurrent glioblastoma multiforme with a liposomally encapsulated replication-incompetent Semliki forest virus vector carrying the human interleukin-12 gene--a phase I/II clinical protocol. J. Neurooncol..

[136] Kramer M.G., Masner M., Casales E., Moreno M., Smerdou C., Chabalgoity J.A. (2015). Neoadjuvant administration of Semliki Forest virus expressing interleukin-12 combined with attenuated Salmonella eradicates breast cancer metastasis and achieves long-term survival in immunocompetent mice. BMC Cancer.

[137] Alkayyal A.A., Tai L.H., Kennedy M.A., de Souza C.T., Zhang J., Lefebvre C., Sahi S., Ananth A.A., Mahmoud A.B., Makrigiannis A.P. (2017). NK-Cell Recruitment Is Necessary for Eradication of Peritoneal Carcinomatosis with an IL12-Expressing Maraba Virus Cellular Vaccine. Cancer Immunol. Res..

[138] Shin E.J., Wanna G.B., Choi B., Aguila D., Ebert O., Genden E.M., Woo S.L. (2007). Interleukin-12 expression enhances vesicular stomatitis virus oncolytic therapy in murine squamous cell carcinoma. Laryngoscope.

[139] Ryapolova A., Minskaia E., Gasanov N., Moroz V., Krapivin B., Egorov A.D., Laktyushkin V., Zhuravleva S., Nagornych M., Subcheva E. (2023). Development of Recombinant Oncolytic rVSV-mIL12-mGMCSF for Cancer Immunotherapy. Int. J. Mol. Sci..

[140] Granot T., Venticinque L., Tseng J.C., Meruelo D. (2011). Activation of cytotoxic and regulatory functions of NK cells by Sindbis viral vectors. PLoS ONE.

[141] Tseng J.C., Hurtado A., Yee H., Levin B., Boivin C., Benet M., Blank S.V., Pellicer A., Meruelo D. (2004). Using sindbis viral vectors for specific detection and suppression of advanced ovarian cancer in animal models. Cancer Res..

[142] Opp S., Hurtado A., Pampeno C., Lin Z., Meruelo D. (2022). Potent and Targeted Sindbis Virus Platform for Immunotherapy of Ovarian Cancer. Cells.

[143] Scherwitzl I., Opp S., Hurtado A.M., Pampeno C., Loomis C., Kannan K., Yu M., Meruelo D. (2020). Sindbis Virus with Anti-OX40 Overcomes the Immunosuppressive Tumor Microenvironment of Low-Immunogenic Tumors. Mol. Ther. Oncolytics.

[144] Triozzi P.L., Strong T.V., Bucy R.P., Allen K.O., Carlisle R.R., Moore S.E., Lobuglio A.F., Conry R.M. (2005). Intratumoral administration of a recombinant canarypox virus expressing interleukin 12 in patients with metastatic melanoma. Hum. Gene Ther..

[145] Triozzi P.L., Allen K.O., Carlisle R.R., Craig M., LoBuglio A.F., Conry R.M. (2005). Phase I study of the intratumoral administration of recombinant canarypox viruses expressing B7.1 and interleukin 12 in patients with metastatic melanoma. Clin. Cancer Res..

[146] Puisieux I., Odin L., Poujol D., Moingeon P., Tartaglia J., Cox W., Favrot M. (1998). Canarypox virus-mediated interleukin 12 gene transfer into murine mammary adenocarcinoma induces tumor suppression and long-term antitumoral immunity. Hum. Gene Ther..

[147] Jiang H., Nace R., Carrasco T.F., Zhang L., Whye Peng K., Russell S.J. (2024). Oncolytic varicella-zoster virus engineered with ORF8 deletion and armed with drug-controllable interleukin-12. J. Immunother. Cancer.

[148] Paunovska K., Loughrey D., Dahlman J.E. (2022). Drug delivery systems for RNA therapeutics. Nat. Rev. Genet..

[149] Aslan C., Kiaie S.H., Zolbanin N.M., Lotfinejad P., Ramezani R., Kashanchi F., Jafari R. (2021). Exosomes for mRNA delivery: A novel biotherapeutic strategy with hurdles and hope. BMC Biotechnol..

[150] Yang Z., Shi J., Xie J., Wang Y., Sun J., Liu T., Zhao Y., Zhao X., Wang X., Ma Y. (2020). Large-scale generation of functional mRNA-encapsulating exosomes via cellular nanoporation. Nat. Biomed. Eng..

[151] You Y., Tian Y., Yang Z., Shi J., Kwak K.J., Tong Y., Estania A.P., Cao J., Hsu W.H., Liu Y. (2023). Intradermally delivered mRNA-encapsulating extracellular vesicles for collagen-replacement therapy. Nat. Biomed. Eng..

[152] Li Y., Ma X., Yue Y., Zhang K., Cheng K., Feng Q., Ma N., Liang J., Zhang T., Zhang L. (2022). Rapid Surface Display of mRNA Antigens by Bacteria-Derived Outer Membrane Vesicles for a Personalized Tumor Vaccine. Adv. Mater..

[153] Gao X., Li Y., Nie G., Zhao X. (2023). mRNA Delivery Platform Based on Bacterial Outer Membrane Vesicles for Tumor Vaccine. Bio Protoc..

[154] Segel M., Lash B., Song J., Ladha A., Liu C.C., Jin X., Mekhedov S.L., Macrae R.K., Koonin E.V., Zhang F. (2021). Mammalian retrovirus-like protein PEG10 packages its own mRNA and can be pseudotyped for mRNA delivery. Science.

[155] Zochowska M., Piguet A.C., Jemielity J., Kowalska J., Szolajska E., Dufour J.F., Chroboczek J. (2015). Virus-like particle-mediated intracellular delivery of mRNA cap analog with in vivo activity against hepatocellular carcinoma. Nanomedicine.

[156] Zhou J., Liu J., Cheng C.J., Patel T.R., Weller C.E., Piepmeier J.M., Jiang Z., Saltzman W.M. (2011). Biodegradable poly(amine-co-ester) terpolymers for targeted gene delivery. Nat. Mater..

[157] Kauffman A.C., Piotrowski-Daspit A.S., Nakazawa K.H., Jiang Y., Datye A., Saltzman W.M. (2018). Tunability of Biodegradable Poly(amine- co-ester) Polymers for Customized Nucleic Acid Delivery and Other Biomedical Applications. Biomacromolecules.

[158] Jiang Y., Gaudin A., Zhang J., Agarwal T., Song E., Kauffman A.C., Tietjen G.T., Wang Y., Jiang Z., Cheng C.J. (2018). A “top-down” approach to actuate poly(amine-co-ester) terpolymers for potent and safe mRNA delivery. Biomaterials.

[159] Patel A.K., Kaczmarek J.C., Bose S., Kauffman K.J., Mir F., Heartlein M.W., DeRosa F., Langer R., Anderson D.G. (2019). Inhaled Nanoformulated mRNA Polyplexes for Protein Production in Lung Epithelium. Adv. Mater..

[160] Yang W., Mixich L., Boonstra E., Cabral H. (2023). Polymer-Based mRNA Delivery Strategies for Advanced Therapies. Adv. Healthc. Mater..

[161] Neshat S.Y., Chan C.H.R., Harris J., Zmily O.M., Est-Witte S., Karlsson J., Shannon S.R., Jain M., Doloff J.C., Green J.J. (2023). Polymeric nanoparticle gel for intracellular mRNA delivery and immunological reprogramming of tumors. Biomaterials.

[162] Sun Y., Yang J., Yang T., Li Y., Zhu R., Hou Y., Liu Y. (2021). Co-delivery of IL-12 cytokine gene and cisplatin prodrug by a polymetformin-conjugated nanosystem for lung cancer chemo-gene treatment through chemotherapy sensitization and tumor microenvironment modulation. Acta Biomater..

[163] Sun Y., Liu L., Zhou L., Yu S., Lan Y., Liang Q., Liu J., Cao A., Liu Y. (2020). Tumor Microenvironment-Triggered Charge Reversal Polymetformin-Based Nanosystem Co-Delivered Doxorubicin and IL-12 Cytokine Gene for Chemo-Gene Combination Therapy on Metastatic Breast Cancer. ACS Appl. Mater. Interfaces.

[164] Estapé Senti M., García Del Valle L., Schiffelers R.M. (2024). mRNA delivery systems for cancer immunotherapy: Lipid nanoparticles and beyond. Adv. Drug Deliv. Rev..

[165] Kon E., Ad-El N., Hazan-Halevy I., Stotsky-Oterin L., Peer D. (2023). Targeting cancer with mRNA-lipid nanoparticles: Key considerations and future prospects. Nat. Rev. Clin. Oncol..

[166] Zong Y., Lin Y., Wei T., Cheng Q. (2023). Lipid Nanoparticle (LNP) Enables mRNA Delivery for Cancer Therapy. Adv. Mater..

[167] Cheng Q., Wei T., Farbiak L., Johnson L.T., Dilliard S.A., Siegwart D.J. (2020). Selective organ targeting (SORT) nanoparticles for tissue-specific mRNA delivery and CRISPR-Cas gene editing. Nat. Nanotechnol..

[168] Dilliard S.A., Cheng Q., Siegwart D.J. (2021). On the mechanism of tissue-specific mRNA delivery by selective organ targeting nanoparticles. Proc. Natl. Acad. Sci. USA.

[169] Lai I., Swaminathan S., Baylot V., Mosley A., Dhanasekaran R., Gabay M., Felsher D.W. (2018). Lipid nanoparticles that deliver IL-12 messenger RNA suppress tumorigenesis in MYC oncogene-driven hepatocellular carcinoma. J. Immunother. Cancer.

[170] Hewitt S.L., Bailey D., Zielinski J., Apte A., Musenge F., Karp R., Burke S., Garcon F., Mishra A., Gurumurthy S. (2020). Intratumoral IL12 mRNA Therapy Promotes TH1 Transformation of the Tumor Microenvironment. Clin. Cancer Res..

[171] Luo M., Liang X., Luo S.T., Wei X.W., Liu T., Ren J., Ma C.C., Yang Y.H., Wang B.L., Liu L. (2015). Folate-Modified Lipoplexes Delivering the Interleukin-12 Gene for Targeting Colon Cancer Immunogene Therapy. J. Biomed. Nanotechnol..

[172] Jia S.F., Worth L.L., Densmore C.L., Xu B., Duan X., Kleinerman E.S. (2003). Aerosol gene therapy with PEI: IL-12 eradicates osteosarcoma lung metastases. Clin. Cancer Res..

[173] Rodrigo-Garzón M., Berraondo P., Ochoa L., Zulueta J.J., González-Aseguinolaza G. (2010). Antitumoral efficacy of DNA nanoparticles in murine models of lung cancer and pulmonary metastasis. Cancer Gene Ther..

[174] Jia S.F., Worth L.L., Densmore C.L., Xu B., Zhou Z., Kleinerman E.S. (2002). Eradication of osteosarcoma lung metastases following intranasal interleukin-12 gene therapy using a nonviral polyethylenimine vector. Cancer Gene Ther..

[175] Duan X., Jia S.F., Koshkina N., Kleinerman E.S. (2006). Intranasal interleukin-12 gene therapy enhanced the activity of ifosfamide against osteosarcoma lung metastases. Cancer.

[176] Maheshwari A., Mahato R.I., McGregor J., Han S., Samlowski W.E., Park J.S., Kim S.W. (2000). Soluble biodegradable polymer-based cytokine gene delivery for cancer treatment. Mol. Ther..

[177] Son H.J., Kim J.S. (2007). Therapeutic efficacy of DNA-loaded PLGA microspheres in tumor-bearing mice. Arch. Pharm. Res..

[178] Maheshwari A., Han S., Mahato R.I., Kim S.W. (2002). Biodegradable polymer-based interleukin-12 gene delivery: Role of induced cytokines, tumor infiltrating cells and nitric oxide in anti-tumor activity. Gene Ther..

[179] Mahato R.I., Lee M., Han S., Maheshwari A., Kim S.W. (2001). Intratumoral delivery of p2CMVmIL-12 using water-soluble lipopolymers. Mol. Ther..

[180] Janát-Amsbury M.M., Yockman J.W., Lee M., Kern S., Furgeson D.Y., Bikram M., Kim S.W. (2005). Local, non-viral IL-12 gene therapy using a water soluble lipopolymer as carrier system combined with systemic paclitaxel for cancer treatment. J. Control Release.

[181] Janát-Amsbury M.M., Yockman J.W., Lee M., Kern S., Furgeson D.Y., Bikram M., Kim S.W. (2004). Combination of local, nonviral IL12 gene therapy and systemic paclitaxel treatment in a metastatic breast cancer model. Mol. Ther..

[182] Yockman J.W., Maheshwari A., Han S.O., Kim S.W. (2003). Tumor regression by repeated intratumoral delivery of water soluble lipopolymers/p2CMVmIL-12 complexes. J. Control Release.

[183] Wang Y., Gao S., Ye W.H., Yoon H.S., Yang Y.Y. (2006). Co-delivery of drugs and DNA from cationic core-shell nanoparticles self-assembled from a biodegradable copolymer. Nat. Mater..

[184] Kim T.H., Jin H., Kim H.W., Cho M.H., Cho C.S. (2006). Mannosylated chitosan nanoparticle-based cytokine gene therapy suppressed cancer growth in BALB/c mice bearing CT-26 carcinoma cells. Mol. Cancer Ther..

[185] Sonabend A.M., Velicu S., Ulasov I.V., Han Y., Tyler B., Brem H., Matar M.M., Fewell J.G., Anwer K., Lesniak M.S. (2008). A safety and efficacy study of local delivery of interleukin-12 transgene by PPC polymer in a model of experimental glioma. Anticancer. Drugs.

[186] Díez S., Navarro G., de Ilarduya C.T. (2009). In vivo targeted gene delivery by cationic nanoparticles for treatment of hepatocellular carcinoma. J. Gene Med..

[187] Shen H.H., Peng J.F., Wang R.R., Wang P.Y., Zhang J.X., Sun H.F., Liang Y., Li Y.M., Xue J.N., Li Y.J. (2024). IL-12-Overexpressed Nanoparticles Suppress the Proliferation of Melanoma Through Inducing ICD and Activating DC, CD8(+) T, and CD4(+) T Cells. Int. J. Nanomed..

[188] Chen P., Yang W., Nagaoka K., Huang G.L., Miyazaki T., Hong T., Li S., Igarashi K., Takeda K., Kakimi K. (2023). An IL-12-Based Nanocytokine Safely Potentiates Anticancer Immunity through Spatiotemporal Control of Inflammation to Eradicate Advanced Cold Tumors. Adv. Sci..

[189] Li J., Lin W., Chen H., Xu Z., Ye Y., Chen M. (2020). Dual-target IL-12-containing nanoparticles enhance T cell functions for cancer immunotherapy. Cell Immunol..

[190] Liu J.Q., Zhang C., Zhang X., Yan J., Zeng C., Talebian F., Lynch K., Zhao W., Hou X., Du S. (2022). Intratumoral delivery of IL-12 and IL-27 mRNA using lipid nanoparticles for cancer immunotherapy. J. Control Release.

[191] Wang Z., Chen Y., Wu H., Wang M., Mao L., Guo X., Zhu J., Ye Z., Luo X., Yang X. (2024). Intravenous administration of IL-12 encoding self-replicating RNA-lipid nanoparticle complex leads to safe and effective antitumor responses. Sci. Rep..

[192] Li Y., Su Z., Zhao W., Zhang X., Momin N., Zhang C., Wittrup K.D., Dong Y., Irvine D.J., Weiss R. (2020). Multifunctional oncolytic nanoparticles deliver self-replicating IL-12 RNA to eliminate established tumors and prime systemic immunity. Nat. Cancer.

[193] Zhao P., Tian Y., Lu Y., Zhang J., Tao A., Xiang G., Liu Y. (2022). Biomimetic calcium carbonate nanoparticles delivered IL-12 mRNA for targeted glioblastoma sono-immunotherapy by ultrasound-induced necroptosis. J. Nanobiotechnol..

[194] Liu H., Du Y., Zhan D., Yu W., Li Y., Wang A., Yin J., Cao H., Fu Y. (2024). Oxaliplatin lipidated prodrug synergistically enhances the anti-colorectal cancer effect of IL12 mRNA. Drug Deliv. Transl. Res..

[195] Xu S., Xu Y., Solek N.C., Chen J., Gong F., Varley A.J., Golubovic A., Pan A., Dong S., Zheng G. (2024). Tumor-Tailored Ionizable Lipid Nanoparticles Facilitate IL-12 Circular RNA Delivery for Enhanced Lung Cancer Immunotherapy. Adv. Mater..

[196] Tros De Ilarduya C., Buñuales M., Qian C., Düzgüneş N. (2006). Antitumoral activity of transferrin-lipoplexes carrying the IL-12 gene in the treatment of colon cancer. J. Drug Target..

[197] Men K., Huang R., Zhang X., Zhang R., Zhang Y., He M., Tong R., Yang L., Wei Y., Duan X. (2018). Local and Systemic Delivery of Interleukin-12 Gene by Cationic Micelles for Cancer Immunogene Therapy. J. Biomed. Nanotechnol..

[198] Charoensit P., Kawakami S., Higuchi Y., Yamashita F., Hashida M. (2010). Enhanced growth inhibition of metastatic lung tumors by intravenous injection of ATRA-cationic liposome/IL-12 pDNA complexes in mice. Cancer Gene Ther..

[199] Liu M., Hu S., Yan N., Popowski K.D., Cheng K. (2024). Inhalable extracellular vesicle delivery of IL-12 mRNA to treat lung cancer and promote systemic immunity. Nat. Nanotechnol..

[200] Zhang J., Song H., Dong Y., Li G., Li J., Cai Q., Yuan S., Wang Y., Song H. (2023). Surface Engineering of HEK293 Cell-Derived Extracellular Vesicles for Improved Pharmacokinetic Profile and Targeted Delivery of IL-12 for the Treatment of Hepatocellular Carcinoma. Int. J. Nanomed..

[201] Rossowska J., Anger N., Wegierek K., Szczygieł A., Mierzejewska J., Milczarek M., Szermer-Olearnik B., Pajtasz-Piasecka E. (2019). Antitumor Potential of Extracellular Vesicles Released by Genetically Modified Murine Colon Carcinoma Cells with Overexpression of Interleukin-12 and shRNA for TGF-β1. Front. Immunol..

[202] Lewis N.D., Sia C.L., Kirwin K., Haupt S., Mahimkar G., Zi T., Xu K., Dooley K., Jang S.C., Choi B. (2021). Exosome Surface Display of IL12 Results in Tumor-Retained Pharmacology with Superior Potency and Limited Systemic Exposure Compared with Recombinant IL12. Mol. Cancer Ther..

[203] Barnwal A., Ganguly S., Bhattacharyya J. (2023). Multifaceted Nano-DEV-IL for Sustained Release of IL-12 to Avert the Immunosuppressive Tumor Microenvironment and IL-12-Associated Toxicities. ACS Appl. Mater. Interfaces.

[204] Stephan M.T., Moon J.J., Um S.H., Bershteyn A., Irvine D.J. (2010). Therapeutic cell engineering with surface-conjugated synthetic nanoparticles. Nat. Med..

[205] Huang B., Abraham W.D., Zheng Y., Bustamante López S.C., Luo S.S., Irvine D.J. (2015). Active targeting of chemotherapy to disseminated tumors using nanoparticle-carrying T cells. Sci. Transl. Med..

[206] Zhang W., Wang M., Tang W., Wen R., Zhou S., Lee C., Wang H., Jiang W., Delahunty I.M., Zhen Z. (2022). Nanoparticle-Laden Macrophages for Tumor-Tropic Drug Delivery. Adv. Mater..

[207] Siriwon N., Kim Y.J., Siegler E., Chen X., Rohrs J.A., Liu Y., Wang P. (2018). CAR-T Cells Surface-Engineered with Drug-Encapsulated Nanoparticles Can Ameliorate Intratumoral T-cell Hypofunction. Cancer Immunol. Res..

[208] Hato L., Vizcay A., Eguren I., Pérez-Gracia J.L., Rodríguez J., Gállego Pérez-Larraya J., Sarobe P., Inogés S., Díaz de Cerio A.L., Santisteban M. (2024). Dendritic Cells in Cancer Immunology and Immunotherapy. Cancers.

[209] Komita H., Zhao X., Katakam A.K., Kumar P., Kawabe M., Okada H., Braughler J.M., Storkus W.J. (2009). Conditional interleukin-12 gene therapy promotes safe and effective antitumor immunity. Cancer Gene Ther..

[210] Akiyama Y., Watanabe M., Maruyama K., Ruscetti F.W., Wiltrout R.H., Yamaguchi K. (2000). Enhancement of antitumor immunity against B16 melanoma tumor using genetically modified dendritic cells to produce cytokines. Gene Ther..

[211] Yoshida M., Jo J., Tabata Y. (2010). Augmented anti-tumor effect of dendritic cells genetically engineered by interleukin-12 plasmid DNA. J. Biomater. Sci. Polym. Ed..

[212] Yao W., Li Y., Zeng L., Zhang X., Zhou Z., Zheng M., Wan H. (2019). Intratumoral injection of dendritic cells overexpressing interleukin-12 inhibits melanoma growth. Oncol. Rep..

[213] Zhao X., Bose A., Komita H., Taylor J.L., Kawabe M., Chi N., Spokas L., Lowe D.B., Goldbach C., Alber S. (2011). Intratumoral IL-12 gene therapy results in the crosspriming of Tc1 cells reactive against tumor-associated stromal antigens. Mol. Ther..

[214] Okada N., Iiyama S., Okada Y., Mizuguchi H., Hayakawa T., Nakagawa S., Mayumi T., Fujita T., Yamamoto A. (2005). Immunological properties and vaccine efficacy of murine dendritic cells simultaneously expressing melanoma-associated antigen and interleukin-12. Cancer Gene Ther..

[215] Rodríguez-Calvillo M., Duarte M., Tirapu I., Berraondo P., Mazzolini G., Qian C., Prieto J., Melero I. (2002). Upregulation of natural killer cells functions underlies the efficacy of intratumorally injected dendritic cells engineered to produce interleukin-12. Exp. Hematol..

[216] Mierzejewska J., Węgierek-Ciura K., Rossowska J., Szczygieł A., Anger-Góra N., Szermer-Olearnik B., Geneja M., Pajtasz-Piasecka E. (2022). The Beneficial Effect of IL-12 and IL-18 Transduced Dendritic Cells Stimulated with Tumor Antigens on Generation of an Antitumor Response in a Mouse Colon Carcinoma Model. J. Immunol. Res..

[217] Melero I., Duarte M., Ruiz J., Sangro B., Galofré J., Mazzolini G., Bustos M., Qian C., Prieto J. (1999). Intratumoral injection of bone-marrow derived dendritic cells engineered to produce interleukin-12 induces complete regression of established murine transplantable colon adenocarcinomas. Gene Ther..

[218] Tatsumi T., Huang J., Gooding W.E., Gambotto A., Robbins P.D., Vujanovic N.L., Alber S.M., Watkins S.C., Okada H., Storkus W.J. (2003). Intratumoral delivery of dendritic cells engineered to secrete both interleukin (IL)-12 and IL-18 effectively treats local and distant disease in association with broadly reactive Tc1-type immunity. Cancer Res..

[219] Tatsumi T., Takehara T., Yamaguchi S., Sasakawa A., Miyagi T., Jinushi M., Sakamori R., Kohga K., Uemura A., Ohkawa K. (2007). Injection of IL-12 gene-transduced dendritic cells into mouse liver tumor lesions activates both innate and acquired immunity. Gene Ther..

[220] Shimizu T., Berhanu A., Redlinger R.E., Watkins S., Lotze M.T., Barksdale E.M. (2001). Interleukin-12 transduced dendritic cells induce regression of established murine neuroblastoma. J. Pediatr. Surg..

[221] Saika T., Satoh T., Kusaka N., Ebara S., Mouraviev V.B., Timme T.L., Thompson T.C. (2004). Route of administration influences the antitumor effects of bone marrow-derived dendritic cells engineered to produce interleukin-12 in a metastatic mouse prostate cancer model. Cancer Gene Ther..

[222] Huang C., Ramakrishnan R., Trkulja M., Ren X., Gabrilovich D.I. (2012). Therapeutic effect of intratumoral administration of DCs with conditional expression of combination of different cytokines. Cancer Immunol. Immunother..

[223] Mazzolini G., Alfaro C., Sangro B., Feijoó E., Ruiz J., Benito A., Tirapu I., Arina A., Sola J., Herraiz M. (2005). Intratumoral injection of dendritic cells engineered to secrete interleukin-12 by recombinant adenovirus in patients with metastatic gastrointestinal carcinomas. J. Clin. Oncol..

[224] Di Trani C.A., Cirella A., Arrizabalaga L., Bella Á., Fernandez-Sendin M., Russo-Cabrera J.S., Gomar C., Olivera I., Bolaños E., González-Gomariz J. (2023). Intracavitary adoptive transfer of IL-12 mRNA-engineered tumor-specific CD8(+) T cells eradicates peritoneal metastases in mouse models. Oncoimmunology.

[225] Etxeberria I., Bolaños E., Quetglas J.I., Gros A., Villanueva A., Palomero J., Sánchez-Paulete A.R., Piulats J.M., Matias-Guiu X., Olivera I. (2019). Intratumor Adoptive Transfer of IL-12 mRNA Transiently Engineered Antitumor CD8(+) T Cells. Cancer Cell.

[226] Olivera I., Bolaños E., Gonzalez-Gomariz J., Hervas-Stubbs S., Mariño K.V., Luri-Rey C., Etxeberria I., Cirella A., Egea J., Glez-Vaz J. (2023). mRNAs encoding IL-12 and a decoy-resistant variant of IL-18 synergize to engineer T cells for efficacious intratumoral adoptive immunotherapy. Cell Rep. Med..

[227] Chinnasamy D., Yu Z., Kerkar S.P., Zhang L., Morgan R.A., Restifo N.P., Rosenberg S.A. (2012). Local delivery of interleukin-12 using T cells targeting VEGF receptor-2 eradicates multiple vascularized tumors in mice. Clin. Cancer Res..

[228] Kerkar S.P., Leonardi A.J., van Panhuys N., Zhang L., Yu Z., Crompton J.G., Pan J.H., Palmer D.C., Morgan R.A., Rosenberg S.A. (2013). Collapse of the tumor stroma is triggered by IL-12 induction of Fas. Mol. Ther..

[229] Chmielewski M., Kopecky C., Hombach A.A., Abken H. (2011). IL-12 release by engineered T cells expressing chimeric antigen receptors can effectively Muster an antigen-independent macrophage response on tumor cells that have shut down tumor antigen expression. Cancer Res..

[230] Meister H., Look T., Roth P., Pascolo S., Sahin U., Lee S., Hale B.D., Snijder B., Regli L., Ravi V.M. (2022). Multifunctional mRNA-Based CAR T Cells Display Promising Antitumor Activity Against Glioblastoma. Clin. Cancer Res..

[231] Pegram H.J., Lee J.C., Hayman E.G., Imperato G.H., Tedder T.F., Sadelain M., Brentjens R.J. (2012). Tumor-targeted T cells modified to secrete IL-12 eradicate systemic tumors without need for prior conditioning. Blood.

[232] Kueberuwa G., Kalaitsidou M., Cheadle E., Hawkins R.E., Gilham D.E. (2018). CD19 CAR T Cells Expressing IL-12 Eradicate Lymphoma in Fully Lymphoreplete Mice through Induction of Host Immunity. Mol. Ther. Oncolytics.

[233] Koneru M., Purdon T.J., Spriggs D., Koneru S., Brentjens R.J. (2015). IL-12 secreting tumor-targeted chimeric antigen receptor T cells eradicate ovarian tumors in vivo. Oncoimmunology.

[234] Liu Y., Di S., Shi B., Zhang H., Wang Y., Wu X., Luo H., Wang H., Li Z., Jiang H. (2019). Armored Inducible Expression of IL-12 Enhances Antitumor Activity of Glypican-3-Targeted Chimeric Antigen Receptor-Engineered T Cells in Hepatocellular Carcinoma. J. Immunol..

[235] Luo Y., Chen Z., Sun M., Li B., Pan F., Ma A., Liao J., Yin T., Tang X., Huang G. (2022). IL-12 nanochaperone-engineered CAR T cell for robust tumor-immunotherapy. Biomaterials.

[236] Yang Z., Pietrobon V., Bobbin M., Stefanson O., Yang J., Goswami A., Alphson B., Choi H., Magallanes K., Cai Q. (2023). Nanoscale, antigen encounter-dependent, IL-12 delivery by CAR T cells plus PD-L1 blockade for cancer treatment. J. Transl. Med..

[237] Kułach N., Pilny E., Cichoń T., Czapla J., Jarosz-Biej M., Rusin M., Drzyzga A., Matuszczak S., Szala S., Smolarczyk R. (2021). Mesenchymal stromal cells as carriers of IL-12 reduce primary and metastatic tumors of murine melanoma. Sci. Rep..

[238] Park J., Park S.A., Kim Y.-S., Kim D., Shin S., Lee S.H., Jeun S.-S., Chung Y.-J., Ahn S. (2024). Intratumoral IL-12 delivery via mesenchymal stem cells combined with PD-1 blockade leads to long-term antitumor immunity in a mouse glioblastoma model. Biomed. Pharmacother..

[239] Elzaouk L., Moelling K., Pavlovic J. (2006). Anti-tumor activity of mesenchymal stem cells producing IL-12 in a mouse melanoma model. Exp. Dermatol..

[240] McKenna M.K., Englisch A., Brenner B., Smith T., Hoyos V., Suzuki M., Brenner M.K. (2021). Mesenchymal stromal cell delivery of oncolytic immunotherapy improves CAR-T cell antitumor activity. Mol. Ther..

[241] Eliopoulos N., Francois M., Boivin M.N., Martineau D., Galipeau J. (2008). Neo-organoid of marrow mesenchymal stromal cells secreting interleukin-12 for breast cancer therapy. Cancer Res..

[242] Hong X., Miller C., Savant-Bhonsale S., Kalkanis S.N. (2009). Antitumor treatment using interleukin- 12-secreting marrow stromal cells in an invasive glioma model. Neurosurgery.

[243] Gao P., Ding Q., Wu Z., Jiang H., Fang Z. (2010). Therapeutic potential of human mesenchymal stem cells producing IL-12 in a mouse xenograft model of renal cell carcinoma. Cancer Lett..

[244] Jeong K.Y., Lee E.J., Kim S.J., Yang S.H., Sung Y.C., Seong J. (2015). Irradiation-induced localization of IL-12-expressing mesenchymal stem cells to enhance the curative effect in murine metastatic hepatoma. Int. J. Cancer.

[245] Wu J., Xie S., Li H., Zhang Y., Yue J., Yan C., Liu K., Liu Y., Xu R., Zheng G. (2021). Antitumor effect of IL-12 gene-modified bone marrow mesenchymal stem cells combined with Fuzheng Yiliu decoction in an in vivo glioma nude mouse model. J. Transl. Med..

[246] Zhang H., Feng Y., Xie X., Song T., Yang G., Su Q., Li T., Li S., Wu C., You F. (2022). Engineered Mesenchymal Stem Cells as a Biotherapy Platform for Targeted Photodynamic Immunotherapy of Breast Cancer. Adv. Healthc. Mater..

[247] Hombach A.A., Geumann U., Günther C., Hermann F.G., Abken H. (2020). IL7-IL12 Engineered Mesenchymal Stem Cells (MSCs) Improve A CAR T Cell Attack Against Colorectal Cancer Cells. Cells.

[248] Seo S.H., Kim K.S., Park S.H., Suh Y.S., Kim S.J., Jeun S.S., Sung Y.C. (2011). The effects of mesenchymal stem cells injected via different routes on modified IL-12-mediated antitumor activity. Gene Ther..

[249] Ryu C.H., Park S.H., Park S.A., Kim S.M., Lim J.Y., Jeong C.H., Yoon W.S., Oh W.I., Sung Y.C., Jeun S.S. (2011). Gene therapy of intracranial glioma using interleukin 12-secreting human umbilical cord blood-derived mesenchymal stem cells. Hum. Gene Ther..

[250] Asada H., Kishida T., Hirai H., Satoh E., Ohashi S., Takeuchi M., Kubo T., Kita M., Iwakura Y., Imanishi J. (2002). Significant antitumor effects obtained by autologous tumor cell vaccine engineered to secrete interleukin (IL)-12 and IL-18 by means of the EBV/lipoplex. Mol. Ther..

[251] Satoh T., Saika T., Ebara S., Kusaka N., Timme T.L., Yang G., Wang J., Mouraviev V., Cao G., el Fattah M.A. (2003). Macrophages transduced with an adenoviral vector expressing interleukin 12 suppress tumor growth and metastasis in a preclinical metastatic prostate cancer model. Cancer Res..

[252] Tabata K., Watanabe M., Naruishi K., Edamura K., Satoh T., Yang G., Abdel Fattah E., Wang J., Goltsov A., Floryk D. (2009). Therapeutic effects of gelatin matrix-embedded IL-12 gene-modified macrophages in a mouse model of residual prostate cancer. Prostate Cancer Prostatic Dis..

[253] Landoni E., Woodcock M.G., Barragan G., Casirati G., Cinella V., Stucchi S., Flick L.M., Withers T.A., Hudson H., Casorati G. (2024). IL-12 reprograms CAR-expressing natural killer T cells to long-lived Th1-polarized cells with potent antitumor activity. Nat. Commun..

[254] Croce M., Meazza R., Orengo A.M., Radić L., De Giovanni B., Gambini C., Carlini B., Pistoia V., Mortara L., Accolla R.S. (2005). Sequential immunogene therapy with interleukin-12- and interleukin-15-engineered neuroblastoma cells cures metastatic disease in syngeneic mice. Clin. Cancer Res..

[255] Galvan D.L., O’Neil R.T., Foster A.E., Huye L., Bear A., Rooney C.M., Wilson M.H. (2015). Anti-Tumor Effects after Adoptive Transfer of IL-12 Transposon-Modified Murine Splenocytes in the OT-I-Melanoma Mouse Model. PLoS ONE.

[256] ClinicalTrials.gov (2021). National Institutes of Health Clinical Center. NHS-IL12 for Solid Tumors. https://clinicaltrials.gov/study/NCT01417546?cond=NCT01417546&rank=1.

[257] Strauss J., Heery C.R., Kim J.W., Jochems C., Donahue R.N., Montgomery A.S., McMahon S., Lamping E., Marté J.L., Madan R.A. (2019). First-in-Human Phase I Trial of a Tumor-Targeted Cytokine (NHS-IL12) in Subjects with Metastatic Solid Tumors. Clin. Cancer Res..

[258] ClinicalTrials.gov (2023). Charalampos Floudas, M., DMSc, MS, National Cancer Institute. Combination Immunotherapy in Subjects with Advanced HPV Associated Malignancies. https://clinicaltrials.gov/study/NCT04287868?cond=NCT04287868&rank=1.

[259] ClinicalTrials.gov (2024). Jason Redman, N.C.I. Phase II Trial of Combination Immunotherapy in Subjects with Advanced Small Bowel and Colorectal Cancers. https://clinicaltrials.gov/study/NCT04491955?cond=https://clinicaltrials.gov/study/NCT04491955?cond=.

[260] ClinicalTrials.gov (2024). EMD Serono (EMD Serono Research & Development Institute. A Phase Ib Study to Evaluate the Safety, Tolerability, and Pharmacokinetics (PK) of Avelumab in Combination with M9241(NHS-IL12) (JAVELIN IL-12) (COMBO). https://clinicaltrials.gov/study/NCT02994953?cond=NCT02994953&rank=1.

[261] ClinicalTrials.gov (2024). National Institutes of Health Clinical Center. NHS-IL12 Monotherapy and in Combination with M7824 in Advanced Kaposi Sarcoma. https://clinicaltrials.gov/study/NCT04303117?cond=NCT04303117&rank=1.

[262] ClinicalTrials.gov (2024). National Institutes of Health Clinical Center. Bintrafusp Alfa (M7824) and NHS-IL12 (M9241) Alone and in Combination with Stereotactic Body Radiation Therapy (SBRT) in Adults with Metastatic Non-Prostate Genitourinary Malignancies. https://clinicaltrials.gov/study/NCT04235777?cond=NCT04235777&rank=1.

[263] ClinicalTrials.gov (2024). Farzan Siddiqui, H.F.H.S. Phase 1 Trial of Interleukin 12 Gene Therapy for Locally Recurrent Prostate Cancer. https://clinicaltrials.gov/study/NCT02555397?cond=NCT02555397&rank=1.

[264] ClinicalTrials.gov (2008). Baylor College of Medicine. Vector Delivery of the IL-12 Gene in Men with Prostate Cancer. https://clinicaltrials.gov/study/NCT00406939?cond=NCT00406939&rank=1..

[265] ClinicalTrials.gov (2022). David Kwon, M., Henry Ford Health System. Phase 1 Trial of Interleukin 12 Gene Therapy for Metastatic Pancreatic Cancer. https://clinicaltrials.gov/study/NCT03281382?cond=NCT03281382&rank=1.

[266] ClinicalTrials.gov (2017). Max Sung, Icahn School of Medicine at Mount Sinai. Gene Therapy in Treating Women with Metastatic Breast Cancer. https://clinicaltrials.gov/study/NCT00849459?cond=NCT00849459&rank=1.

[267] ClinicalTrials.gov (2017). Max Sung, Icahn School of Medicine at Mount Sinai. Biological Therapy in Treating Women with Breast Cancer That Has Spread to the Liver. https://clinicaltrials.gov/study/NCT00301106?cond=NCT00301106&rank=1.

[268] ClinicalTrials.gov (2015). Alaunos Therapeutics. Safety Study of Adenovirus Vector Engineered to Express hIL-12 in Combination with Activator Ligand to Treat Melanoma. https://clinicaltrials.gov/study/NCT01397708?cond=NCT01397708&rank=1.

[269] ClinicalTrials.gov (2013). National Cancer Institute. Vaccine Therapy in Treating Patients with Melanoma. https://clinicaltrials.gov/study/NCT00003556?cond=NCT00003556&rank=1.

[270] ClinicalTrials.gov (2021). Alaunos Therapeutics. A Study of Ad-RTS-hIL-12 + Veledimex in Pediatric Subjects with Brain Tumors Including DIPG. https://clinicaltrials.gov/study/NCT03330197?cond=NCT03330197&rank=1.

[271] ClinicalTrials.gov (2021). Alaunos Therapeutics. A Study of Ad-RTS-hIL-12 with Veledimex in Subjects with Glioblastoma or Malignant Glioma. https://clinicaltrials.gov/study/NCT02026271?cond=NCT02026271&rank=1.

[272] ClinicalTrials.gov (2021). Alaunos Therapeutics. A Study of Ad-RTS-hIL-12 with Veledimex in Combination with Nivolumab in Subjects with Glioblastoma; a Substudy to ATI001-102. https://clinicaltrials.gov/study/NCT03636477?cond=NCT03636477&rank=1.

[273] ClinicalTrials.gov (2024). ames Markert, MD, University of Alabama at Birmingham. Study of Pembrolizumab and M032 (NSC 733972). https://clinicaltrials.gov/study/NCT05084430?cond=NCT05084430&rank=1.

[274] ClinicalTrials.gov (2024). AstraZeneca. A Study of MEDI9253 in Combination with Durvalumab in Select Solid Tumors. https://clinicaltrials.gov/study/NCT04613492?cond=NCT04613492&rank=1.

[275] Petry H., Brooks A., Orme A., Wang P., Liu P., Xie J., Kretschmer P., Qian H.S., Hermiston T.W., Harkins R.N. (2008). Effect of viral dose on neutralizing antibody response and transgene expression after AAV1 vector re-administration in mice. Gene Ther..

[276] Goswami R., Subramanian G., Silayeva L., Newkirk I., Doctor D., Chawla K., Chattopadhyay S., Chandra D., Chilukuri N., Betapudi V. (2019). Gene Therapy Leaves a Vicious Cycle. Front. Oncol..

[277] Wang Y., Hu J.K., Krol A., Li Y.P., Li C.Y., Yuan F. (2003). Systemic dissemination of viral vectors during intratumoral injection. Mol. Cancer Ther..

[278] Egilmez N.K., Jong Y.S., Iwanuma Y., Jacob J.S., Santos C.A., Chen F.A., Mathiowitz E., Bankert R.B. (1998). Cytokine immunotherapy of cancer with controlled release biodegradable microspheres in a human tumor xenograft/SCID mouse model. Cancer Immunol. Immunother..

[279] Sharma A., Harper C.M., Hammer L., Nair R.E., Mathiowitz E., Egilmez N.K. (2004). Characterization of cytokine-encapsulated controlled-release microsphere adjuvants. Cancer Biother. Radiopharm..

[280] Cerqueira B.B., Lasham A., Shelling A.N., Al-Kassas R. (2015). Nanoparticle therapeutics: Technologies and methods for overcoming cancer. Eur. J. Pharm. Biopharm..

[281] Zu H., Gao D. (2021). Non-viral Vectors in Gene Therapy: Recent Development, Challenges, and Prospects. Aaps J.

